# Red-to-Blue Triplet–Triplet Annihilation Upconversion for Calcium Sensing

**DOI:** 10.1021/acs.jpclett.4c01528

**Published:** 2024-07-15

**Authors:** Valeriia D. Andreeva, Irene Regeni, Tingxiang Yang, Anna Elmanova, Martin Presselt, Benjamin Dietzek-Ivanšić, Sylvestre Bonnet

**Affiliations:** † Leiden Institute of Chemistry, Leiden University, Einsteinweg 55, 2333 CC Leiden, Netherlands; ‡ School of BiosciencesUniversity of Sheffield, Alfred Denny Building, Western Bank, Sheffield S10 2TN, United Kingdom; § Leibniz Institute of Photonic Technology, Albert-Einstein-Straße 9, 07745 Jena, Germany; ∥ Institute of Physical Chemistry, Friedrich Schiller University Jena, Helmholtzweg 4, 07743 Jena, Germany; ⊥ SciClus GmbH & Co. KG, Moritz-von-Rohr-Straße 1a, 07745 Jena, Germany; # Center for Energy and Environmental Chemistry Jena (CEEC Jena), Friedrich Schiller University Jena, Philosophenweg 7a, 07743 Jena, Germany

## Abstract

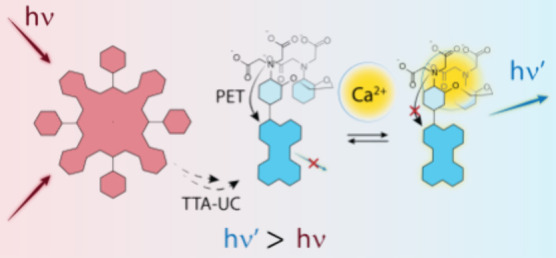

Triplet–triplet annihilation upconversion is a bimolecular process converting low-energy photons into high-energy photons. Here, we report a calcium-sensing system working via triplet–triplet annihilation (TTA) upconverted emission. The probe itself was obtained by covalent conjugation of a blue emitter, perylene, with a calcium-chelating moiety, and it was sensitized by the red-light-absorbing photosensitizer palladium(II) tetraphenyltetrabenzoporphyrin (PdTPTBP). Sensing was selective for Ca^2+^ and occurred in the micromolar domain. In deoxygenated conditions, the TTA upconverted luminescence gradually appeared upon adding an increasing concentration of calcium ions, to reach a maximum upconversion quantum yield of 0.0020.

To study calcium-dependent biological processes,^1−5^ many sensing systems selective to Ca^2+^ have been developed, such as small-molecule,^6−8^ genetically-encoded,^9,10^ and photoacoustic^11,12^ probes. However, application of the existing probes in tissues or organs is limited by light penetration and low contrast as a result of autofluorescence. One approach to overcome these obstacles is to develop sensors with absorption in the red or far-red part of the spectrum (λ_exc_ > 620 nm).^7,13,14^ However, in downconverting sensors, red light excitation means near-infrared emission, i.e., a small energy gap between the highest occupied molecular orbital (HOMO) and the lowest unoccupied molecular orbital (LUMO), which usually results in significant non-radiative decay rates and, hence, a lower difference between the off and on states of the sensor. Another approach that allows overcoming the problems of autofluorescence, limited light penetration, and low contrast between on and off states was chosen in this work: to develop an upconversion mechanism transforming low-energy photons into high-energy photons in a Ca^2+^-dependent manner. Several rare-earth-metal-based upconversion nanoparticles (UCNPs) were proposed to do this.^15−17^ These Ca^2+^-sensitive UCNPs sense the cation irreversibly as a result of the change of the nanoparticle structure upon Ca^2+^ binding, which is applicable for fundamental studies but irrelevant for diagnostic purposes. Here, we report the first calcium sensing system based on the triplet–triplet annihilation upconversion (TTA-UC) mechanism that is capable of transforming red light excitation (630 nm) into anti-Stokes, calcium-dependent blue luminescence. Our system combines TTA-UC and photoinduced electron transfer (PET) mechanisms.

TTA-UC is a bimolecular photophysical process that leads to upconversion with high quantum yields at comparatively low excitation power densities.^18^ A TTA-UC couple includes two types of molecules: a photosensitizer absorbing low-energy photons and an annihilator molecule, which is a fluorophore emitting photons of higher energies (Figure 1). After absorption of a low-energy photon, the photosensitizer generates a triplet excited state *via* intersystem crossing (ISC), followed by triplet–triplet energy transfer (TTET) to the annihilator, to form long-lived annihilator triplet states. Once two annihilator molecules in the triplet states collide, triplet–triplet annihilation (TTA) occurs, resulting in one annihilator molecule reaching a high-energy-emitting singlet excited state, while the second decays to its ground state non-radiatively. Finally, the singlet excited state of the first annihilator emits a photon, thereby realizing upconversion, i.e., anti-Stokes-shifted emission.^18,19^

**Figure 1:**
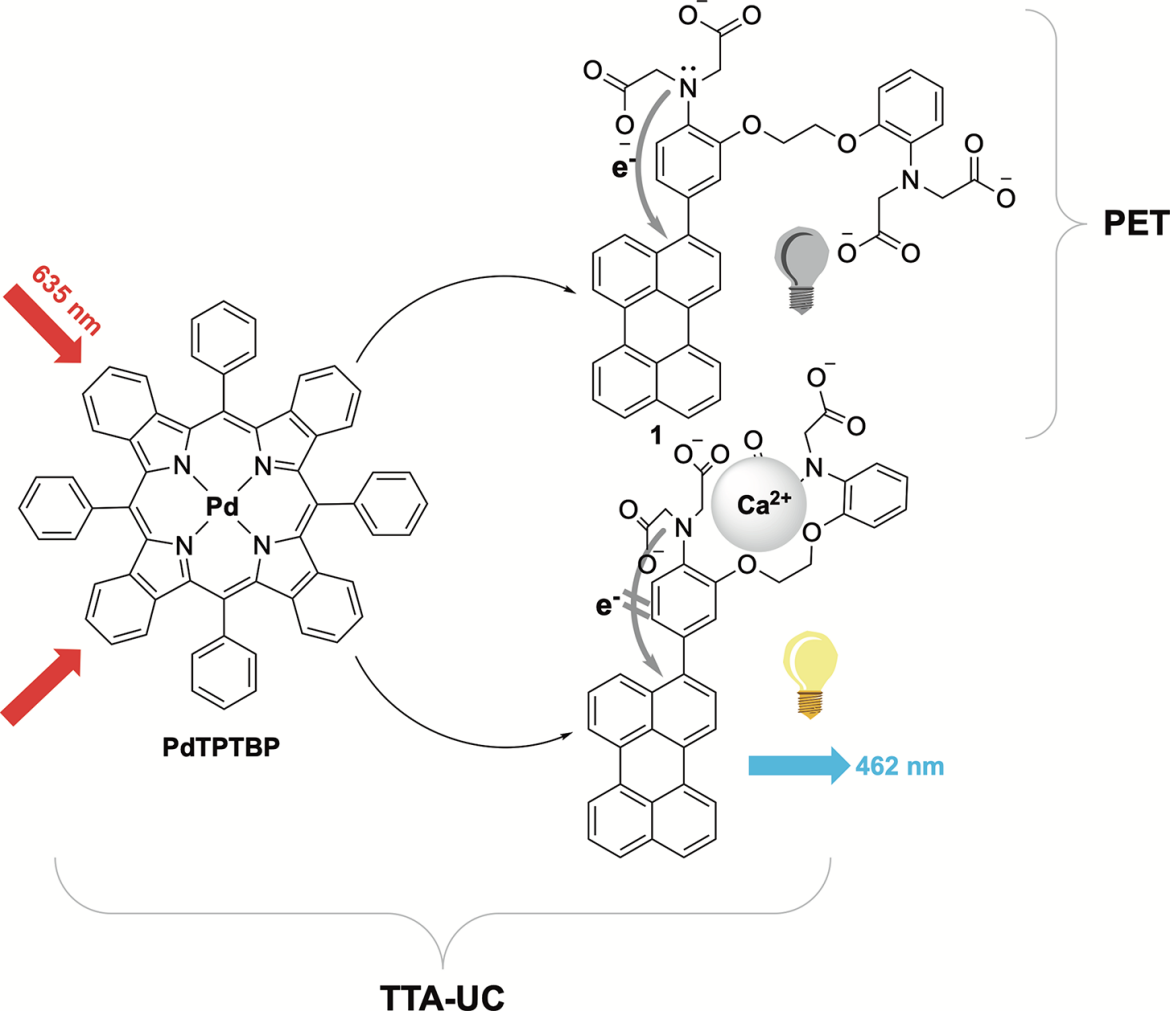
Combining TTA-UC and PET quenching for turn-on calcium ion upconversion sensing.

Several well-characterized TTA-UC couples are known,^20−24^ but examples of TTA-UC application in sensing metal cations are rare and use green-to-blue upconversion. For example, the reported K^+^ turn-off nanosensor utilized a green light sensitizer, PtOEP, along with a blue emitter, diphenylanthracene, mixed within Nile-blue-doped polyvinyl chloride.^25^ Mg^2+^ ions were detected in dichloromethane using green-to-blue TTA-UC, where annihilator emission is quenched, in the absence of Mg^2+^, by PET.^26^ PET quenching occurs when an emitter, in its excited state, is covalently linked to a moiety possessing a high-energy lone pair. One of the electrons of this lone pair transfers to the lowest singly occupied molecular orbital (SOMO) of the excited emitter, before being repleted by the electron in the highest SOMO. Such a scheme results in emission quenching.^27^ Upon cation binding to the chelate, the lone pair will be stabilized, thereby suppressing PET quenching, which switches on upconversion. This approach is one of the most recent methods to apply TTA-UC to sensing.^28^

Red-to-blue upconversion, on the other hand, is more biologically relevant as a result of deeper tissue penetration of red light. One of the rare couples able to do it comprises Pd(II)-*meso*-tetraphenyltetrabenzoporphyrin (PdTPTBP) as a photosensitizer and perylene as a blue-emitting annihilator. In addition to the large anti-Stokes shift of this pair, optimized upconversion quantum yields ranging from 0.05 in toluene or aqueous solutions using liposomes to 0.38 in homogeneous tetrahydrofuran (THF) solution were reported.^20,29^ In this work, we demonstrated that it was possible to realize switch-on sensing of Ca^2+^ ions based on the combination of red-to-blue TTA-UC and PET (Figure 1).

To realize calcium ion sensing via TTA-UC, the calcium-selective ligand^6^ 1,2-bis(*o*-aminophenoxy)ethane-*N*,*N*,*N*′,*N*′-tetraacetic acid (BAPTA, **2**)^6,30,31^ was covalently coupled to a perylene annihilator (**4**) to obtain sensor **1** (Scheme S.2.1 of the Supporting Information). Because the two benzene rings of BAPTA are not conjugated, both mono- and dibromination can occur at the fourth positions of the rings. To specifically obtain the monobromo derivative of BAPTA tetraester (**3**), we conducted the bromination reaction with Br_2_ at −78 °C within 30 s to ensure a rapid reaction and prevent the predominant formation of the dibromo derivative. This reaction produced two products, the dibromo derivative (75% yield) and the monobromo derivative (20% yield), with recovered reagent BAPTA tetraester (25%). Further, the palladium-catalyzed Suzuki cross-coupling reaction between monobrominated BAPTA tetraester **3** and perylene boronic acid **5** led to the acid-protected perylene-based sensor (**6**). Final removal of the ester groups can be performed in different conditions, but purification of the final tetracarboxylate tetrasodium salt was particularly difficult, because it has four different p*K*_a_ values and decarboxylated easily in acidic media [e.g., high-performance liquid chromatography (HPLC)]. Our most successful approach involved subjecting the 98% pure compound **6** to quantitative basic hydrolysis using 5 equiv of NaOH for deprotection. This yielded sensor **1** as a 1:1 mixture of the tetracarboxylate tetrasodium salt and 1 equiv of NaOH, maintaining the solid sample in a basic state. This stable solid could be stored for months in the freezer without undergoing decarboxylation, and its basicity could be neutralized upon dissolution in aqueous buffers. Comprehensive characterization details are provided in Figures S.2.1–S.2.9 of the Supporting Information.

The Ca^2+^-binding properties of sensor **1** were studied with a series of spectroscopic and calorimetric methods, supported by theoretical modeling. First, standard Stokes fluorescence titration of a 6 μM methanol solution of sensor **1** by CaCl_2_·2H_2_O in methanol showed a drastic change in the shape of the emission spectrum, with a decreasing emission around 560 nm, while the 462 nm emission drastically increased upon the addition of Ca^2+^. Plotting the 462 nm fluorescence maximum emission versus the Ca^2+^ concentration showed two consecutive sigmoidal curves representing two binding events (Figure 2a). The fluorescence emission quantum yield of the sensor with 2 equiv of Ca^2+^ was twice higher than that of the calcium-free molecule (0.39 versus 0.18; Table S.5.1 of the Supporting Information). These data were compatible with the design principle of PET quenching in the absence of Ca^2+^, which is suppressed upon calcium binding. The addition of different amounts of CaCl_2_·2H_2_O to a 6 μM sensor **1** solution did not influence the fluorescence lifetimes of the sensor, indicating that static interaction of sensor **1** with Ca^2+^ resulted in suppressed PET in sensor **1** (panels a and b of Figure S.6.1 of the Supporting Information).

**Figure 2:**
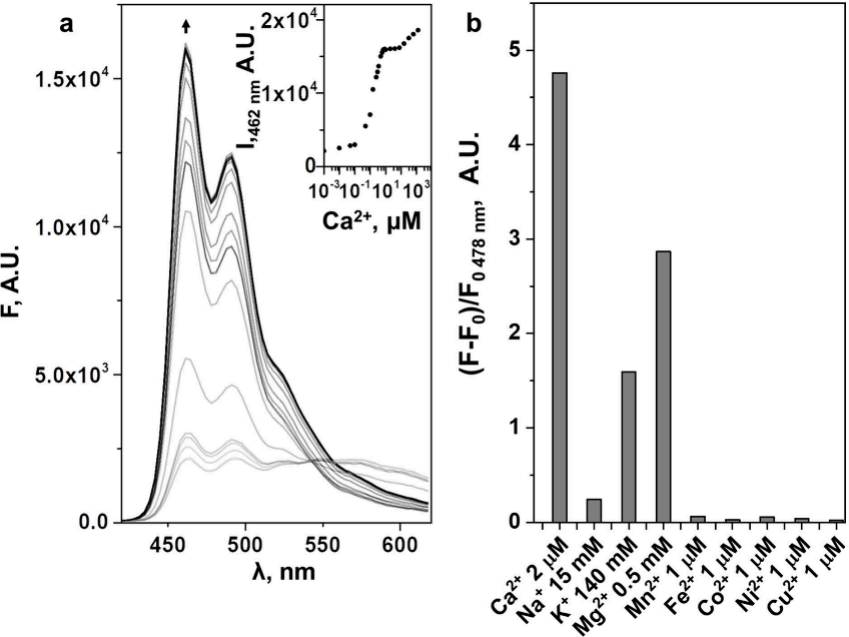
Stokes emission (fluorescence) of sensor **1** in the presence of different metal ions. (a) Titration of sensor **1** (6 μM) with CaCl_2_·2H_2_O in methanol (λ_ex_ = 400 nm and room temperature). (Inset) Plot of the emission maximum of sensor **1** (462 nm) versus Ca^2+^ concentration. (b) Selectivity study: solutions of sensor **1** were prepared in methanol (4 μM, λ_ex_ = 400 nm, and room temperature), after which metal cation concentrations were added, as mentioned in the figure.

The sensor showed selectivity toward Ca^2+^ in methanol solution (4 μM). Figure 2b shows the emission difference comparison at the maximum emission intensity around 465 nm. The addition of 2 μM Ca^2+^ was accompanied by an almost 5-fold increase in emission intensity at 465 nm, while the response to maximal cytosolic concentrations of cell-abundant cations, Na^+^ (15 mM), K^+^ (140 mM), or Mg^2+^ (0.5 mM),^1^ or different first-row transition metals (Fe^2+^, Co^2+^, Ni^2+^, Cu^2+^, and Zn^2+^) at 1 μM concentrations was significantly lower. At 1 μM, however, the response was comparable to the emission increase as a result of the addition of 0.5 mM Mg^2+^. To determine whether Ca^2+^ sensing was possible in presence of 0.5 mM Mg^2+^, we performed a competition experiment (Figure S.5.1 of the Supporting Information). In presence of the maximal cytosolic concentrations of cell-abundant cations, Na^+^ (15 mM), K^+^ (140 mM), or Mg^2+^ (0.5 mM),^1^ or different first-row transition metals (Fe^2+^, Co^2+^, Ni^2+^, Cu^2+^, and Zn^2+^) at 1 μM concentrations, the addition of 1 μM calcium ions was accompanied by a strong increase of the fluorescence of sensor **1**.

Isothermal titration calorimetry (ITC) was further used to investigate the affinity of sensor **1** to Ca^2+^ in methanol and aqueous solution (Figure 3a and Figure S.4.1 of the Supporting Information). Sensor **1** (100 μM) in 10 mM 4-(2-hydroxyethyl)-1-piperazineethanesulfonic acid (HEPES) buffer was first titrated with a 1 mM CaCl_2_·2H_2_O stock solution. Two consecutive binding events were clearly observed in such conditions, of which the association constants were *K*_a1_ = 2.64 ± 0.19 × 10^6^ M^–1^ and *K*_a2_ = 5.05 ± 0.014 × 10^4^ M^–1^, respectively (Figure 3a and Figure S.4.1 and Table S.4.1 of the Supporting Information). Consistently with earlier reports, the first binding event represents the formation of a 1:1 sensor **1**/calcium complex, where the four carboxylate groups and the two amine lone pairs of sensor **1** are coordinated to a single Ca^2+^ cation.^33^ The addition of a second equivalent of Ca^2+^ resulted in a second, to the best of our knowledge, unreported, binding event.

**Figure 3:**
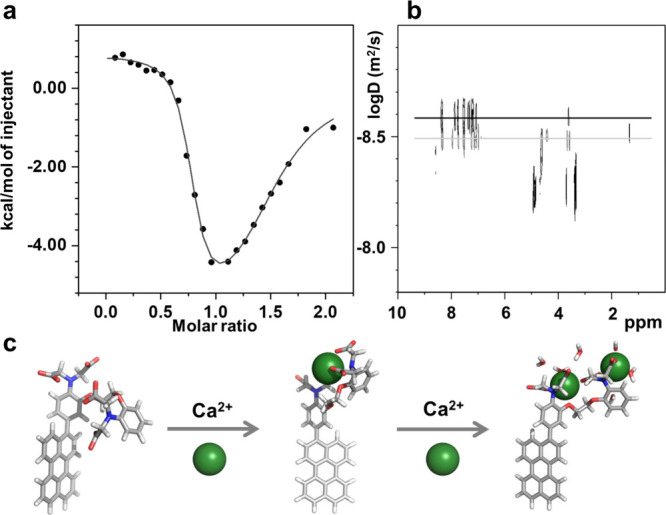
(a) ITC data of sensor **1** (100 μM) titrated with Ca^2+^ solution in HEPES buffer (10 mM, pH 7.2) at 20 °C, (b) ^1^H DOSY NMR of sensor **1**/Ca^2+^ (1:1) solution (black) and a calcium-free sensor **1** in methanol-*d*_4_ (gray) at 298 K, and (c) DFT-optimized models (revPBE0/6-311++/COSMO for a polarizable environment) of sensor **1** in the absence or bound to one or two Ca^2+^.

When the 100 μM solution of sensor **1** was studied in methanol as a solvent instead of the HEPES buffer, a much more complex behavior of the sensor was observed (Figure S.4.2 of the Supporting Information). We were unable to fit those data to 1:1, 1:2, or 2:1 binding models.^34^ However, at millimolar concentrations in methanol-*d*_4_, ^1^H nuclear magnetic resonance (NMR) titration of the sensor with Ca^2+^ ions further supported the initial formation of a 1:1 complex in slow exchange dynamics (Figure S.3.1 of the Supporting Information) until the addition of 1 equiv of Ca^2+^. Upon the addition of 0–1 equiv of Ca^2+^, a new set of signals corresponding to the 1:1 sensor/calcium complex appeared, while the signals of the free sensor **1** disappeared. A further increase of the Ca^2+^ concentration led to sample precipitation, which was consistent with the hypothesis of a 1:2 sensor/calcium complex characterized by a neutral charge and, therefore, reduced solubility in polar solvents.

We assume the second binding event observed by ITC in HEPES or fluorescence spectroscopy in methanol corresponds to the formation of a 1:2 sensor **1**/Ca^2+^ complex, where each biscarboxylatoamine branch of the sensor molecule is involved in the coordination to one Ca^2+^ cation. For this second binding event, we postulate that three solvent molecules saturate the coordination sphere of each metal cations. The density functional theory (DFT) models of sensor **1** with 0, 1, or 2 Ca^2+^ ions per sensor molecule were hence calculated at the revPBE0/6-311++/COSMO level of theory (Figure 3c). According to the DFT-derived energies of these models, the formation of 1:2 adducts was indeed favored by enthalpy compared to the 1:1 adduct (Table S.7.1 of the Supporting Information). ^1^H diffusion-ordered spectroscopy (DOSY) experiments in methanol-*d*_4_ yielded diffusion coefficients and hydrodynamic radii (*r*_H_) in line with the computed sizes of the DFT models of the free sensor and its 1:1 calcium complex (panels b and c of Figure 3 and Figure S.3.2 and Table S.3.2 of the Supporting Information). Overall, sensor **1** was found to strongly bind one Ca^2+^ ion in aqueous or methanol solution and, upon the addition of a second equivalent of Ca^2+^, to lead to complex aggregation and/or precipitation phenomena, depending upon the concentration. We hypothesize that this secondary binding event is the result of the formation of a neutral hydrophobic dicalcium adduct.

In a second series of experiments, sensor **1** was used as annihilator in combination with the red-light-absorbing photosensitizer PdTPTBP, to study TTA-UC in the presence of varying Ca^2+^ concentrations in methanol. First, we studied TTET from the sensitizer to sensor **1** by measuring the evolution of the phosphorescence intensity of PdTPTBP under red light excitation (630 nm) as a function of the concentration of sensor **1** (Figure S.5.3 of the Supporting Information). The phosphorescence intensity decreased upon the addition of sensor **1**, which suggested transfer of the triplet energy of the emitting ^3^PdTPTBP* state to sensor **1** by the Dexter mechanism. The lifetime of the photosensitizer (PS) triplet state was 4 μs (at 5 μM), and that of the annihilator **1** in the absence of calcium was 119 μs (at 75 μM; Figure S.6.2 of the Supporting Information). For this and later experiments, the annihilator concentration was kept low (75 μM) to avoid aggregation. Upon increasing the concentration of sensor **1**, the PS triplet state became shorter lived following a Stern–Volmer relationship (panels a and b of Figure 4). The Stern–Volmer constant *K*_SV_ was 0.424 μM^–1^, which corresponded to a diffusion-limited bimolecular quenching rate constant *k*_TTET_ = 3.8 × 10^9^ M^–1^ s^–1^. Increasing the concentration of Ca^2+^ to 100 μM did not influence the triplet lifetimes of either molecule (Figure S.6.3 of the Supporting Information), which both remained in the same order of magnitude (4 μs for PS and 114 μs for sensor **1**). This result demonstrated that the triplet state of sensor **1** was not influenced by the binding of Ca^2+^ to the BAPTA moiety. Noteworthy, the lifetimes of the triplet state of sensor **1** shortened upon increasing the sensor concentration (Figure 4a). We assume that such shortening correlates with the more efficient TTA process at a higher sensor concentration as a result of the higher probability of two molecules of sensor **1** in the triplet state colliding.

**Figure 4:**
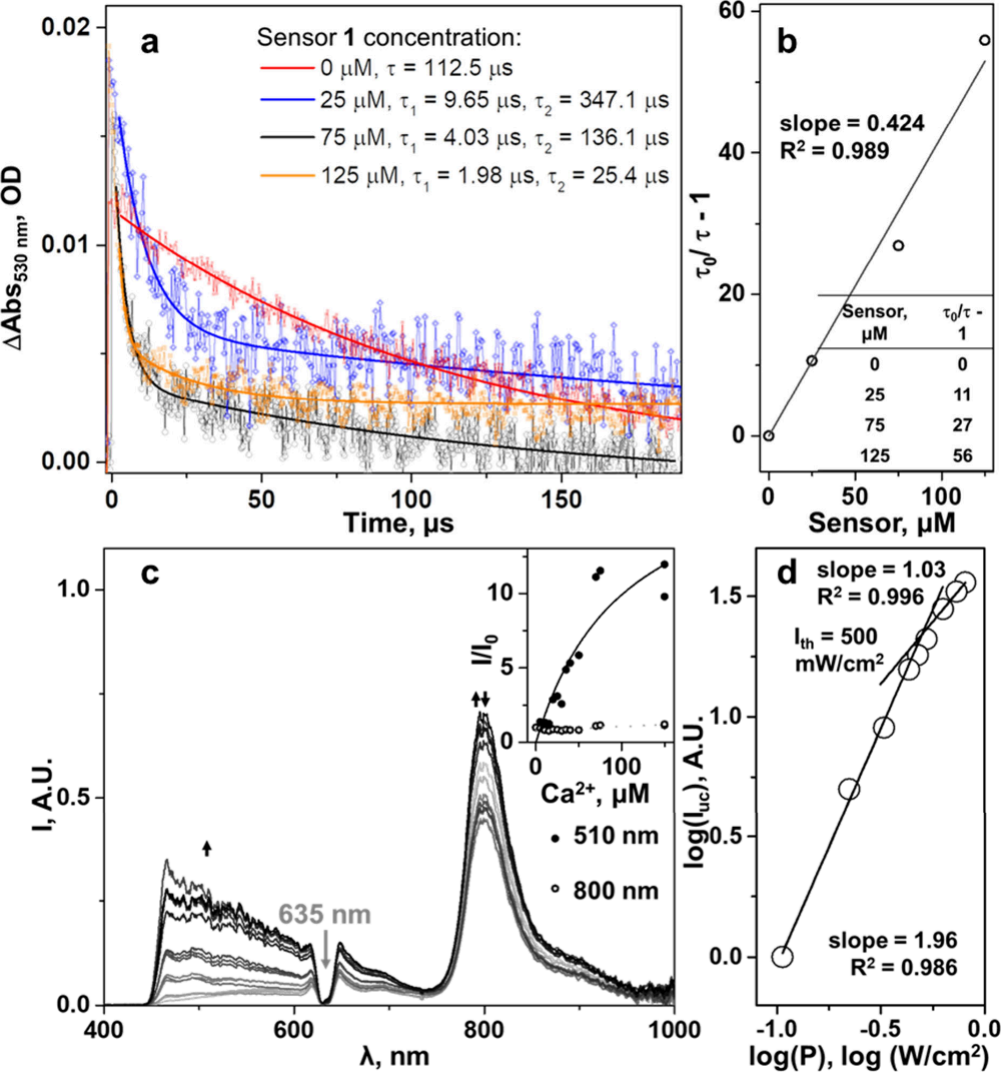
(a) PdTPTBP excited state absorption decays fit at 530 nm wavelength in the presence of different sensor **1** concentrations. Without sensor **1**, we observe a monoexponential decay, which turns into biexponential with addition of the sensor. The first component of the decay shortens with an increase of the sensor **1** concentration (τ_1_). This represents the quenching effect of the sensor on the porphyrin triplet excited state. The second lifetime (τ_2_) is the triplet lifetime of the annihilator. It shortens as a result of the increase of the TTA rate with the increase of the annihilator concentration. (b) Stern–Volmer plot of the PdTPTBP triplet state quenching by sensor **1**. (c) Titration of the PS (2.5 μM) and sensor **1** (75 μM) with Ca^2+^. (Inset) Plot of upconversion luminescence maximum divided by the luminescence maximum without Ca^2+^ (filled dots, fitted with one binding site model) and plotted phosphorescence signal (empty dots). (d) Intensity threshold determination of PdTPTBP (2.5 μM), sensor **1** (75 μM), and Ca^2+^ (75 μM) (λ_ex_ = 635 nm and laser cross-section area = 2.9 mm^2^). All experiments were made in methanol in deoxygenated conditions at room temperature.

The results of time-resolved spectroscopy suggested that the conditions were suitable for the occurrence of calcium-dependent TTA-UC, because efficient intersystem crossing in PdTPTBP combined to fast TTET to sensor **1** at biologically relevant micromolar concentrations, while the lifetime of the triplet state of sensor **1** was long and independent of Ca^2+^.^35^ Indeed, although in the absence of calcium, the steady-state upconverted emission of a mixture of PdTPTBP (2.5 μM) and sensor **1** (75 μM) in methanol under red light irradiation (635 nm) was negligible (panels c and d of Figure 4), upon adding increasing amounts of Ca^2+^, the upconverted luminescence around 500 nm gradually appeared (Figure 4c). Noticeably, the shape of the upconverted emission of the system in the presence of calcium ions differed from that of the classic PdTPTBP–perylene couple (Figure S.5.4 of the Supporting Information). We assume that this effect was caused by the amphiphilic nature of sensor **1**, because its charge changed upon Ca^2+^ binding, resulting into more hydrophobic neutral species prone to aggregation. We suggest that, in this system, PET strongly quenches the singlet emission of sensor **1** from the non-coordinated BAPTA amines in sensor **1** in the absence of calcium, while in the presence of calcium, the singlet emission of the perylene moiety in sensor **1** was recovered upon Ca^2+^ binding to BAPTA. A control experiment consisting of titrating PdTPTBP (2.5 μM) and perylene (75 μM) with Ca^2+^ under identical conditions, supported this hypothesis: the intensity of perylene upconverted emission decreased with the addition of Ca^2+^ as a result of dilution (Figure S.5.4 of the Supporting Information).

Like in all TTA-UC systems, the upconverted emission intensity of the mixture of sensor **1** and PdTPTBP depends upon the excitation intensity, with a quadratic dependence at low excitation intensity (i.e., the low-power regime) and a linear dependence at a high excitation intensity (i.e., the high-power regime). One of the measures of merit of any TTA-UC system is the intensity threshold *I*_th_ defined as the power density at which the two straight lines fitting these two regimes in the log *I*_UC_ versus log *I*_exc_ plot cross each other (Figure 4d).^36,37^*I*_th_ was found to be 500 mW/cm^2^ in this system in methanol; above such power density, for example, at 1500 mW/cm^2^ at 24 °C, the absolute upconversion quantum yield^20^ becomes independent from the excitation intensity, and a value of 0.0020 was found (Figures S.5.6 and S.5.7b and eq S.5.3 of the Supporting Information). For the system of 2.5 μM PdTPTBP and 25 μM sensor **1** in the presence of 75 μM Ca^2+^, the intensity threshold was in the same order of magnitude as for the system above (around 330 mW/cm^2^; Figure S.5.7a of the Supporting Information). The maximum quantum yield obtained for this sample was 0.0010 (Figure S.5.7b of the Supporting Information). Overall, in such conditions, sensor **1** served as annihilator for Ca^2+^ sensing by red-to-blue TTA-UC in homogeneous methanol or aqueous solutions through PET quenching in the singlet state, while the lifetime of the triplet state remained long and essentially calcium-independent.

In conclusion, a TTA-UC turn-on calcium sensing system has been prepared on the basis of the known PdTPTBP sensitizer and the new sensor **1**, which binds up to two Ca^2+^ ions in methanol and aqueous HEPES buffer solution. In this system, red light transforms into blue light. Red light penetrates further into biological tissues than any previously reported upconverting sensing systems based on TTA-UC. The binding of the sensor to its analyte was investigated using ITC, ^1^H-NMR, ^1^H-DOSY, fluorescence steady-state, and time-resolved spectroscopies. The performance of this upconverting sensing system was characterized by an intensity threshold of 500 mW/cm^2^ and an upconversion quantum yield of 0.002 at room temperature in methanol. For real application of this sensor in biology, multiple challenges will have to be overcome, such as the water insolubility of the PdTPTBP photosensitizer, the oxygen sensitivity of the system, which might be addressed using antioxidants,^38^ or the yet unknown intracellular localization of the sensor molecules, which may require future (nano)formulation of the TTA-UC sensing pair, for example, in nanoparticles.^39^ However, sensor **1** represents one of the rare molecular sensors capable, upon binding to a biometal, of giving an upconverted spectroscopic response that no other molecules present in a living cell may be able to give.

## Supporting Information Available

Synthesis, steady and time-resolved spectroscopy data, beam profiling, and details on ITC measurements and DFT models (PDF)
