# Supplementary material for: Red-to-Blue Triplet–Triplet Annihilation Upconversion for Calcium Sensing
Source: J Phys Chem Lett. 2024 Jul 15;15(29):7430–5. doi: 10.1021/acs.jpclett.4c01528 (PMC11284849; doi:10.1021/acs.jpclett.4c01528)
Supplement: Supplementary file 1 — jz4c01528_si_001.pdf [file jz4c01528_si_001.pdf]

Red-to-Blue Triplet-triplet Annihilation Upconversion for Calcium-sensing

Valeriia D. Andreeva[|], Irene Regeni[|], Tingxiang Yang[ǂ,¡], Anna Elmanova[ǂ,‖,§,$], Martin Presselt[ǂ,‖,§,$], Benjamin Dietzek-Ivanšić[ǂ,‖], and Sylvestre A. Bonnet[|,*]

|-Leiden Institute of Chemistry, Leiden University, Einsteinweg 55, 2333 CC Leiden,The Netherlands
ǂ-Leibniz Institute of Photonic Technology, Albert-Einstein-Straße 9 07745 Jena, Germany
¡-University of Sheffield, Alfred Denny Building, University of, Sheffield S10 2TN, United Kingdom
‖-Friedrich Schiller University, Institute of Physical Chemistry, Helmholtzweg 4, 07743 Jena, German
§-sciclus GmbH & Co. KG, Moritz-von-Rohr-Str. 1a, 07745 Jena, Germany
$-Center for Energy and Environmental Chemistry Jena (CEEC Jena), Friedrich Schiller University Jena, Philosophenweg 7a, 07743 Jena, Germany

Table of Contents

Table of Contents 1
1. Synthesis 2
1.1. Br-BAPTA-Et$_4$ (3) 3
1.3. 3-perylene boronic ester (5) 4
1.4. Per-BAPTA-Et$_4$ (6) 5
1.5. Per-BAPTA (1) 6
2. NMR spectroscopy 10
2.1. ^{1}H – NMR titration 10
2.2. ^{1}H-DOSY spectroscopy 10
3. Isothermal calorimetry titration experiments. 12
3.1. In water solution 12
3.2. In methanol 13
4. Steady-state spectroscopy 14
4.1. Fluorescence titration 14
4.2. Fluorescence quantum yield measurements 14
4.3. Selectivity and competition studies 15
4.4. Upconversion emission spectroscopy 15
4.4.1. Upconversion power dependence measurements. 15
4.4.2. Phosphorescence quenching in presence of the annihilator 1 16
4.4.3. Titration of the PS and the sensor 1 solution in methanol in deoxygenated conditions with Ca^{2+} at r.t. 16
4.4.4. Control titration of the PS and perylene solution in methanol in deoxygenated conditions with Ca^{2+} at r.t. 16
4.5. Quantum yield measurements by absolute method 17
4.6. Beam profiling 18
5. Time-resolved spectroscopy 20
5.1. Fluorescence lifetime measurements 20

5.2. Nanosecond transient absorption spectroscopy 20
6. Computational studies 22
References...23

General Methods

Reagents were purchased from Sigma Aldrich, Fisher Scientific, FluoroChem, VWR and TCI. Solvents were used directly if not mentioned otherwise. Dry DMF was obtained as a result of distillation under nitrogen atmosphere followed by settling over activated 3 Å molecular sieves under nitrogen atmosphese for two days. Thin-layer chromatography (TLC) was performed with Merk silica-coated aluminum plates (F60 with F254 indicator). Column chromatography was performed using 60 Å silica gel (0.04 – 0.063 mm, Screening Devices B.V.). NMR spectra were recorded on a Brucker AV-400, Brucker AV-I-500 or Brucker AV-III-600 spectrometer at 298 K. Recorded spectra were analysed with MestReNova software. Electrospray ionization mass spectra (ESI-MS) were recorded with a Thermo Fisher MSQ Plus mass spectrometer with 17-2000 m/z detection range. High resolution mass spectrometry (HRMS) was performed with a Thermo Finnigan LTQ Orbitap with electrospray ionization (ESI) method. Elemental Analysis was performed by the Microanalytical Laboratory Kolbe (Oberhausen, Germany). Analytical LC-MS was made with an analytical NUCLEODUR™ C18 Gravity column (3 µm, 50 x 4.6 mm,; Macherey-Nagel) connected to a Vanquish™ UHPLC system with a Vanquish™ Diode Array detector coupled to a LCQ™ Fleet via ESI (all Thermo Fisher Scientific).

^{1}H-NMR titration was performed with Brucker AV-400 at room temperature. The recorded spectra were analysed with MestReNova software.

^{1}H-DOSY experiments were made with AV-III-600 at 298 K in 3 mm EPR tubes with delays d20 = 90 ms and p30 = 480 µs. Obtained data were processed with Brucker TopSpin software. Diffusion coefficients determined with the Stejskal-Tanner equation fitting of T1/T2 relaxation module analysis of integrated signals.

Binding constants were determined with isothermal titration calorimetry (ITC) using a MicroCal VP-ITC. Titration was performed at 20 °C with 28 injections (2 µL first and 10 µL following). Milli-Q water was used as a reference, reference power was set for 10 µcal/s, initial delay 60 s, stirring speed 307 rpm. Obtained data were processed with MicroCal ITC-ORIGIN Analysis Software.

Fluorescence emission spectra were recorded with HORIBA Aqualog in 1 cm quarz cuvette. Recorded spectra were processed with Origin 2022 software. All another custom-built setups for upconversion measurements described in corresponding sections below.

1. Synthesis

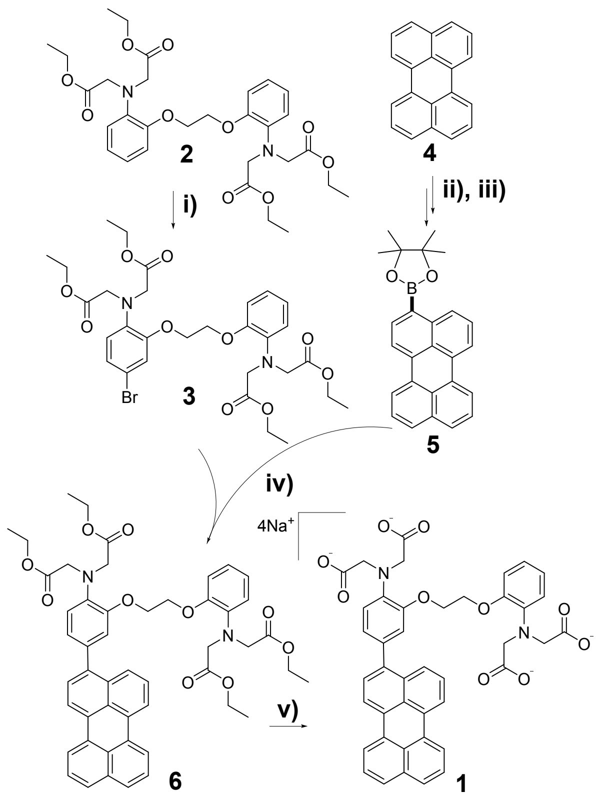

Scheme S.2.1. Synthesis of calcium sensor 1. I) Br_2, Chloroform, -78^{0}C, 20%; ii) NBS, DMF, N_2, r.t.; iii) B_2pin_2, KOAc, 1,4-dioxane, N_2, 70^{0}C, 67%; iv) $Pd(PPh_3)_4$, K_2CO_3, EtOH, toluene, N_2, 100^{0}C, 15%; v) NaOH, H_2O, MeOH, 1,4-dioxane, 40^{0}C, 81%.

1.1. Br-BAPTA-Et$_4$ (3)

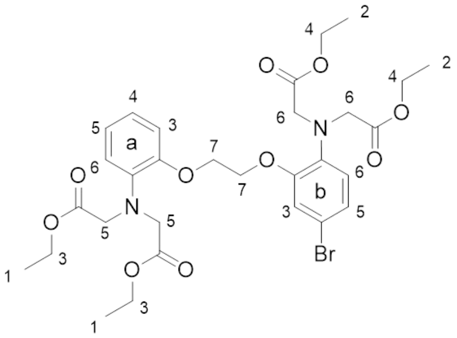

In 10 mL round-bottom flask BAPTA-Et$_4$ (300 mg; 0.51 mmol) was dissolved in freshly distilled chloroform (5 mL). Then pyridine (41 µL, 0.51 mmol) was added to the reaction mixture at once. The obtained solution was cooled down with a liquid nitrogen bath (-196 ^{o}C) till the solvent crystallization. Separately bromine (13 µL, 0.255 mmol) was dissolved in chloroform (1 mL). The bromine solution was then added to the cold reaction mixture. The nitrogen bath was removed to allow the frozen solution to melt. Right after melting of the chloroform, the solution was vigorously stirred for 30 s, after which 2 M HCl was added (5 mL) to quench the reaction. Longer reaction times led invariably to dibromination and lower yields of the final compound. After quenching the reaction mixture was washed with water (1x30 mL) and NaHCO$_3$ sat. (1x30 mL). The organic layer was dried over MgSO$_4$, filtered and evaporated. The crude product was purified with silica column using EtOAc-Pentane (20:80) as eluent, to isolate 78 mg (20%) of the target product **3** and 177 mg (52%) of dibrominated (Br$_2$-BAPTA-Et$_4$).

^{1}H NMR (400 MHz, CDCl$_3$) δ 6.95 (ap. dd, *J* = 8.3, 2.2 Hz, 1H, H^{b5}), 6.92 (ap.d, *J* = 2.2 Hz, 1H, H^{b6}), 6.90 – 6.76 (m, 4H, H^{a3-6}), 6.65 (d, *J* = 8.3 Hz, 1H, H^{a3}), 4.22 (tq, *J* = 5.3, 2.3 Hz, 4H, H^7), 4.11 (s, 4H, H^6), 4.07 (s, 4H, H^5), 4.01 (dq, *J* = 14.3, 7.2 Hz, 8H, H^3+H^4), 1.11 (dt, *J* = 8.7, 7.2 Hz, 12H, H^1+H^2). ^{13}C NMR (101 MHz, CDCl$_3$) δ 171.56, 171.26, 150.96, 150.19, 139.46, 138.62, 124.13, 122.14, 121.64, 120.01, 118.98, 116.17, 113.89, 113.35, 67.73, 67.46, 67.20, 66.93, 60.88, 60.80, 53.48, 53.41, 14.05, 14.01. ESI-MS (m/z) exp. (calc.): 667.3; (667.5 [M+H]$^+$).

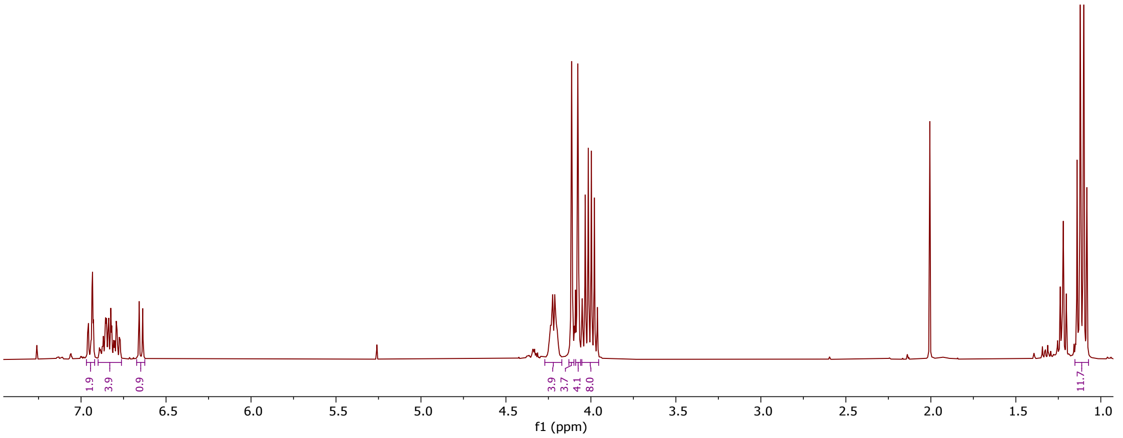

FigureS.2.1. ^{1}H – NMR of compound **3** in CDCl$_3$.

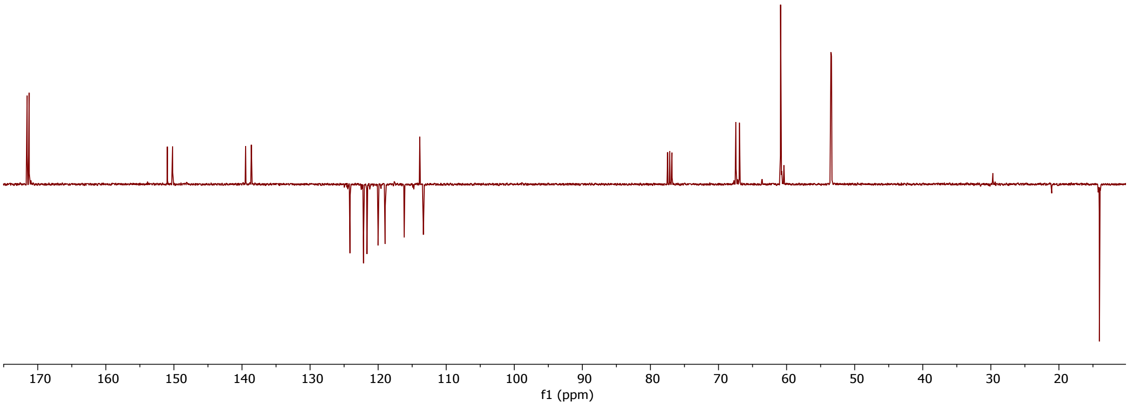

Figure S.2.2. ^{13}C – NMR of compound **3** in CDCl$_3$.

1.2. 3-perylenebromide (4)

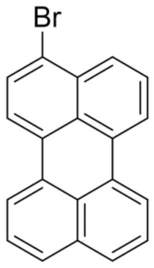

In a 1000 mL two-necked flask equipped with a stirring bar, perylene (1.00 g, 3.96 mmol) was dissolved in dry DMF (180 mL). A solution of N-bromosuccinimide (NBS, 705 mg, 1.98 mmol) in dry DMF (40 mL) was added to the reaction mixture. The solution was stirred for 26 h under nitrogen atmosphere at room temperature. Afterwards water (400 mL) was added to the reaction mixture, which was stirred for 1 h. The precipitated product was filtered, washed with water (250 mL), and redissolved in DCM (500 mL). The obtained organic solution was dried over $MgSO_4$, filtered and rotary evaporated. The crude product, which was a mixture of monobrominated and unbrominated perylene with ratio of 1:0.15, was used in further reactions without additional purification.

^{1}H NMR (500 MHz, $CDCl_3$) δ 8.25 – 8.14 (m, 3H), 8.08 (dd, *J* = 8.3z, 1.0 Hz, 1H), 7.99 (d, *J* = 8.1 Hz, 1H), 7.76 (d, *J* = 8.1 Hz, 1H), 7.71 (ddd, *J* = 8.3, 1.7, 1.0 Hz, 2H), 7.58 (dd, *J* = 8.3, 7.5 Hz, 1H), 7.48 (ddd, *J* = 8.2, 7.5, 6.2 Hz, 2H). Obtained spectrum corresponds to the literature data. [1]

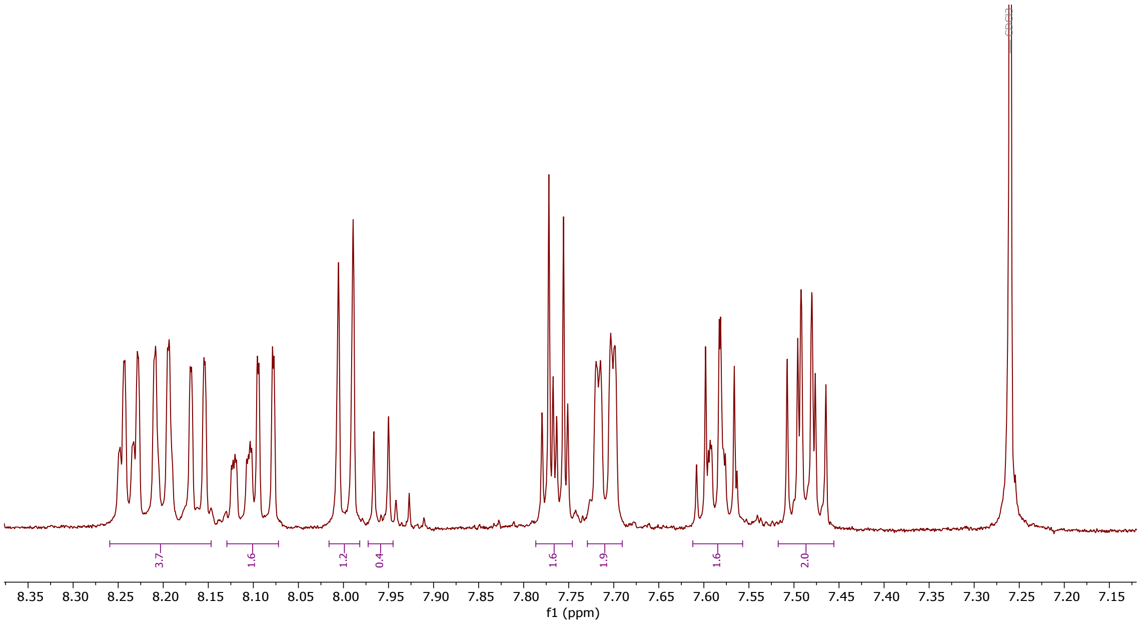

Figure S.2.3. ^{1}H – NMR of the result mixture of compound **4** and perylene in $CDCl_3$.

1.3. 3-perylene boronic ester (5)

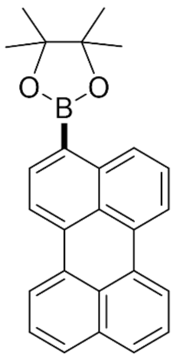

In a 500 mL two-necked round-bottom flask equipped with stirring bar and a reflux condenser, 3-perylenebromide (1.81 g, 5.46 mmol) as a solid mixture with perylene, potassium acetate (1.56 g, 16.4 mmol), and bis(pinacolato)diboron (3.86 g, 15.3 mmol) were dissolved in 1,4-dioxane (200 mL) and purged with nitrogen. Then 1,1'-bis(diphenylphosphino)ferrocene-palladium(II) dichloride (280 mg, 0.383 mmol) was added to the reaction mixture. The reaction mixture was stirred at 70 °C under nitrogen atmosphere overnight. After cooling to room temperature, the solvent was removed under reduced pressure. The residue was extracted with dichloromethane (1x200 mL), washed with sat. $NaHCO_3$ (aq.) (2x200 mL) and brine (1x200 mL). The organic layer was dried over magnesium sulfate, filtered, and dried under reduced pressure. The product was isolated by column chromatography on silica gel using a dichloromethane / hexane (v/v = 1:1) eluent giving 1.38 g (3.65 mmol, 67% yield) of compound **5**.

^{1}H NMR (500 MHz, $CDCl_3$) δ 8.66 (dd, *J* = 8.3, 1.0 Hz, 1H), 8.24 (dd, *J* = 7.6, 1.0 Hz, 1H), 8.21 (ddd, *J* = 7.6, 5.1, 1.1 Hz, 2H), 8.18 (d, *J* = 7.6 Hz, 1H), 8.06 (d, *J* = 7.6 Hz, 1H), 7.71 (dd, *J* = 8.0, 1.0 Hz, 1H), 7.68 (dd, *J* = 8.0, 1.3 Hz, 1H), 7.54 (dd, *J* = 8.3, 7.5 Hz, 1H), 7.49 (td, *J* = 8.1, 7.5, 1.3 Hz, 2H), 1.44 (s, 12H). Spectrum corresponds to the literature data [1].

1.4. Per-BAPTA-Et_4 (6)

In 50 mL round-bottom flask equipped with stirring bar and a Din-Stark cap, 3-perylene boronic ester **5** (373 mg, 0.99 mmol) and Br-BAPTA-Et_4 **3** (330 mg, 0.49 mmol) were dissolved in toluene (16 mL) and ethanol (3 mL). The solution was purged with nitrogen during

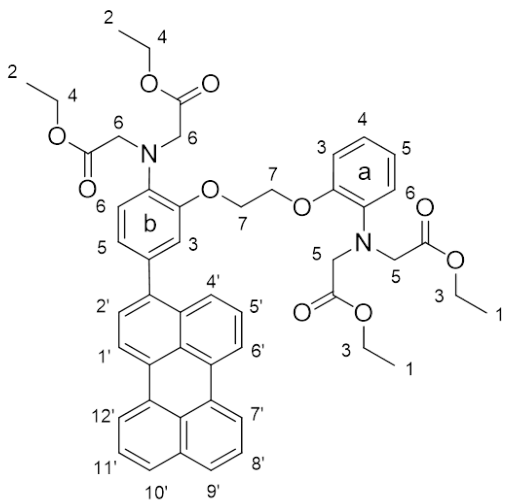

30 min. Then, $Pd(PPh_3)_4$ (57 mg, 0.05 mmol) was added to the solution followed by 2 M aq. K_2CO_3 (0.5 mL). The reaction was stirred at 85 ^{o}C degrees for 20 min. Afterwards the temperature was raised to 100 ^{o}C and the mixture was stirred for 22 h more. After cooling to room temperature, the reaction mixture was washed with water (3x30 mL) and the organic layer was dried over anhydrous Na_2SO_4. After evaporation, ethanol (5 mL) was added to the residue and the precipitate was filtered, washed with ethanol (2x5 mL) and ether (2x5 mL). The crude product was purified by silica gel column chromatography using DCM/EtOAc/triethylamine (v/v, 9/1/0.01) as eluent, followed by preparative TLC in the same eluent to give 66 mg of the title compound **6** (0.493 mmol, 15 %).

^{1}H NMR (400 MHz, $CDCl_3$) δ 8.20 (dd, J = 7.5, 3.3 Hz, 4H, $H^{1',6', 7', 12'}$), 7.81 (dd, J = 8.4, 0.9 Hz, 1H, $H^{4'}$), 7.67 (br. d, J = 8.0 Hz, 2H, $H^{9',10'}$), 7.48 (td, J = 7.8, 1.9 Hz, 2H, $H^{8',11'}$), 7.40 (ap. dd, J = 7.6, 0.8 Hz, 1H, $H^{5'}$), 7.38 (ap. d, J = 7.6 Hz, 1H, $H^{2'}$), 7.05 (ap. dd, J = 8.1, 1.7 Hz, 1H, H^{b5}), 7.02 (ap. d, J = 1.76 Hz, 1H, H^{b3}), 6.92 (d, J = 8.1 Hz, 1H, H^{b6}), 6.90 – 6.79 (m, 4H, $H^{a3\text{-}6}$), 4.34 – 4.27 (m, 4H, H^{7}), 4.26 (s, 4H, H^{6}), 4.15 (s, 4H, H^{5}), 4.06 (dq, J = 17.7, 7.1 Hz, 8H, $H^{3,4}$), 1.18 (t, J = 7.1 Hz, 6H, H^{2}), 1.13 (t, J = 7.1 Hz, 6H, H^{1}). ^{13}C NMR (101 MHz, $CDCl_3$) δ 171.74, 171.71, 150.39, 150.09, 139.77, 139.52, 138.75, 134.80, 134.51, 133.11, 131.54, 131.50, 131.39, 130.49, 129.19, 128.75, 127.88, 127.79, 127.66, 126.74, 126.72, 126.58, 126.21, 123.07, 122.24, 121.60, 120.46, 120.42, 120.15, 120.02, 119.02, 118.52, 114.73, 113.23, 67.25, 67.14, 61.00, 60.86, 53.68, 53.59, 14.17. ESI-MS (m/z) exp. (calc.): 839.3 (839.3 $[M+H]^{+}$), 861.3 (861.3 $[M+Na]^{+}$).

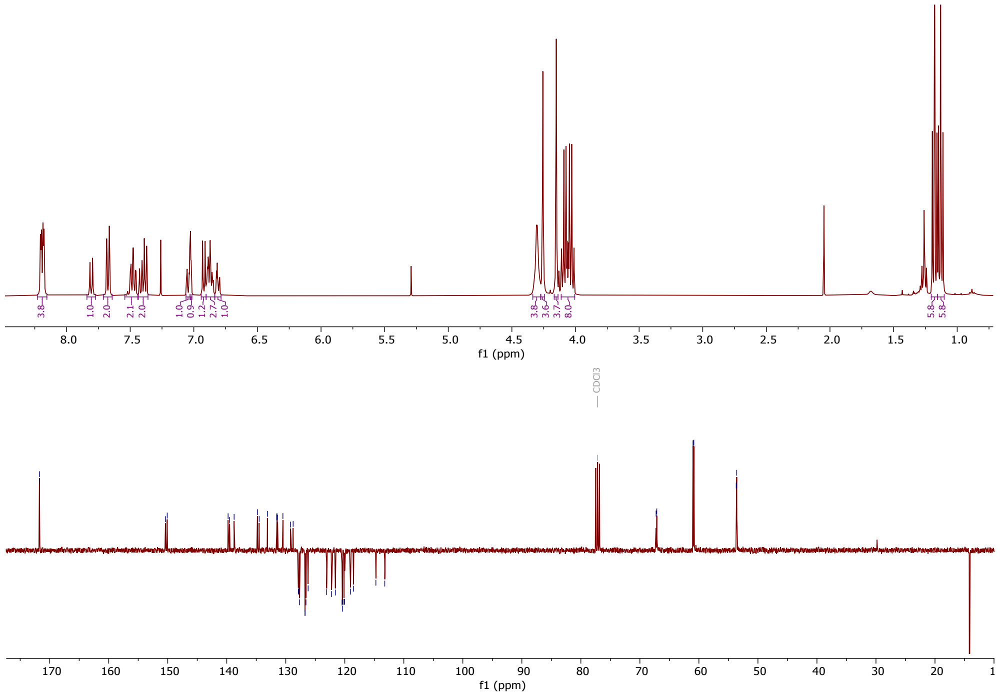

Figure S.2.4. ^{1}H and ^{13}C-NMR of compound **6** in $CDCl_3$, respectively.

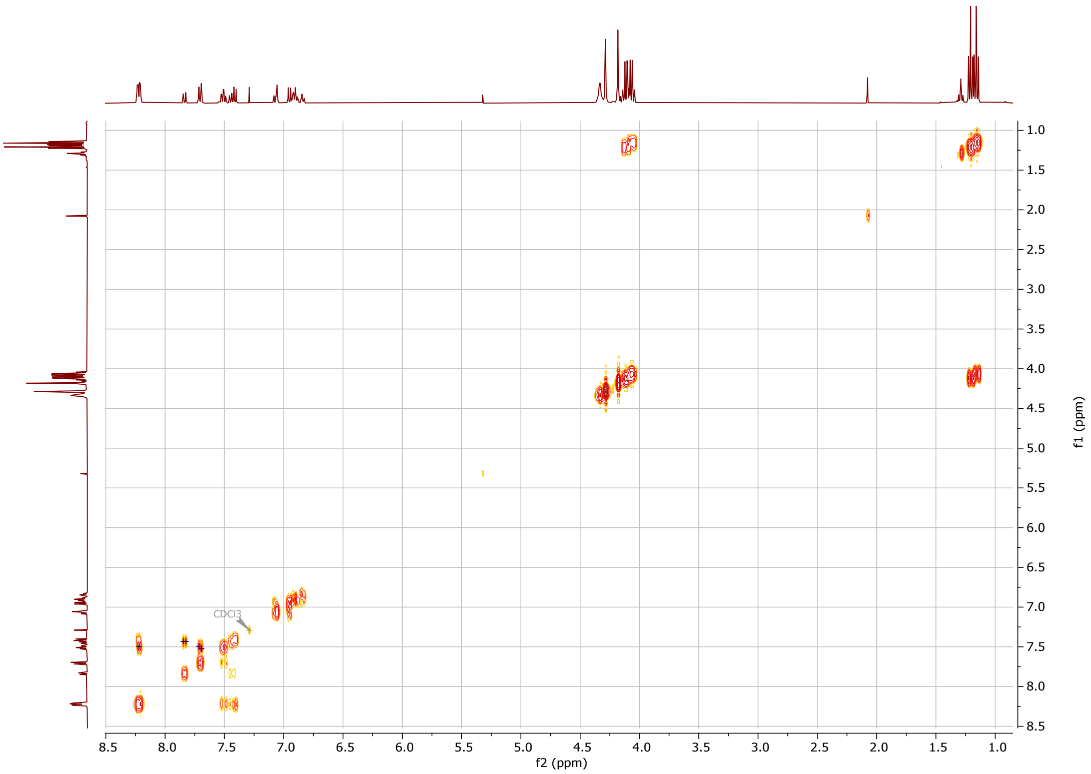

Figure S.2.5. ^{1}H-COSY-NMR of compound **6** in $CDCl_3$.

1.5. Per-BAPTA (1)

In a 25 mL round-bottom flask Per-BAPTA-Et$_4$ **6** (284 mg, 0.34 mmol) was dissolved in a 1,4-dioxane – methanol mixture (6 mL, 14/5 v/v). Separately, 4 N aq. NaOH (0.4 mL, 1.69 mmol, 5 eq.) was mixed with the above-mentioned 1,4-dioxane – methanol mixture (1.6 mL). The

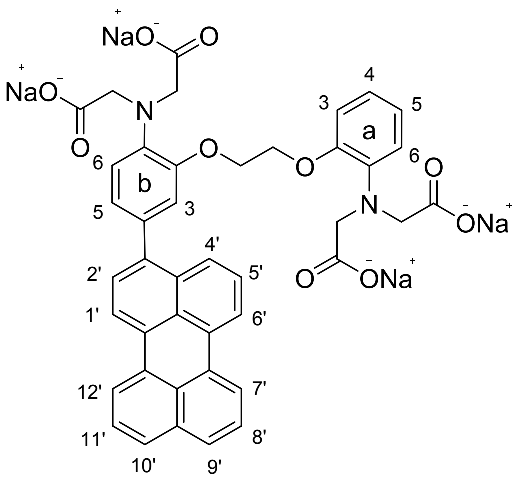

obtained NaOH solution was added to the reaction mixture, which was stirred vigorously at room temperature.Reaction was monitored by an-HPLC. As soon as the peak of the starting compound had disappeared, the reaction was stopped, first by rotary evaporation of the organic solvents, then freeze-drying the water phase. 200 mg (81 %) of the target compound was obtained as a tetrasodium salt containing 1 eq. of NaOH. This compound was pure enough according to HPLC (15 min linear gradient from 10% CH_3CN/0.1%TFA aq. to 90% CH_3CN/0.1%TFA aq.), to be used directly. It must be kept cold and it is sensitive to acids, which decompose it by decarboxylation.

^{1}H NMR (400 MHz, MeOD) δ 8.30 – 8.21 (m, 4H, $H^{1',3',7',12'}$), 7.88 (d, J = 8.5 Hz, 1H, $H^{4'}$), 7.66 (d, J = 8.1 Hz, 2H, $H^{9',10'}$), 7.48 – 7.40 (m, 4H, $H^{2',5',8',11'}$), 7.17 (d, J = 8.1 Hz, 1H, H^{b6}), 7.06 (d, J = 1.9 Hz, 1H, H^{b3}), 7.03 (dd, J = 7.7, 1.3 Hz, 1H, H^{a5}), 7.00 (dd, J = 8.1, 1.8 Hz, 1H, H^{b5}), 6.91 (ddd, J = 7.8, 3.8, 1.3 Hz, 2H, $H^{a3,4}$), 6.86 – 6.79 (m, 1H, H^{a6}), 4.38 – 4.29 (m, 4H, O-C$\underline{H_2}$), 3.59 (s, 4H, N^b-C$\underline{H_2}$), 3.50 (s, 4H, N^a-C$\underline{H_2}$). ^{13}C NMR (101 MHz, MeOD) δ = 179.19, 179.10, 152.38, 152.22, 141.75, 141.31, 141.28, 136.46, 136.26, 134.27, 132.66, 132.55, 132.49, 132.42, 131.50, 129.81, 128.90, 128.81, 128.73, 127.80, 127.68, 127.43, 123.89, 123.35, 121.82, 121.54, 121.46, 121.23, 121.11, 118.92, 118.83, 113.89, 112.10, 84.35, 84.12, 67.25, 67.02, 59.10. ESI-MS (m/z) exp. (calc.): 727.07 (727.1 $[M+H]^+$). Elemental analysis calc. (%) for $C_{42}H_{30}N_2Na_4O_{10}$ + NaOH: C 59.09, H 3.54, N 3.28, Na 13.47; found: C 58.74, H 3.61, N 3.24, Na 13.36. LC-MS: r.t. 8.35 min.

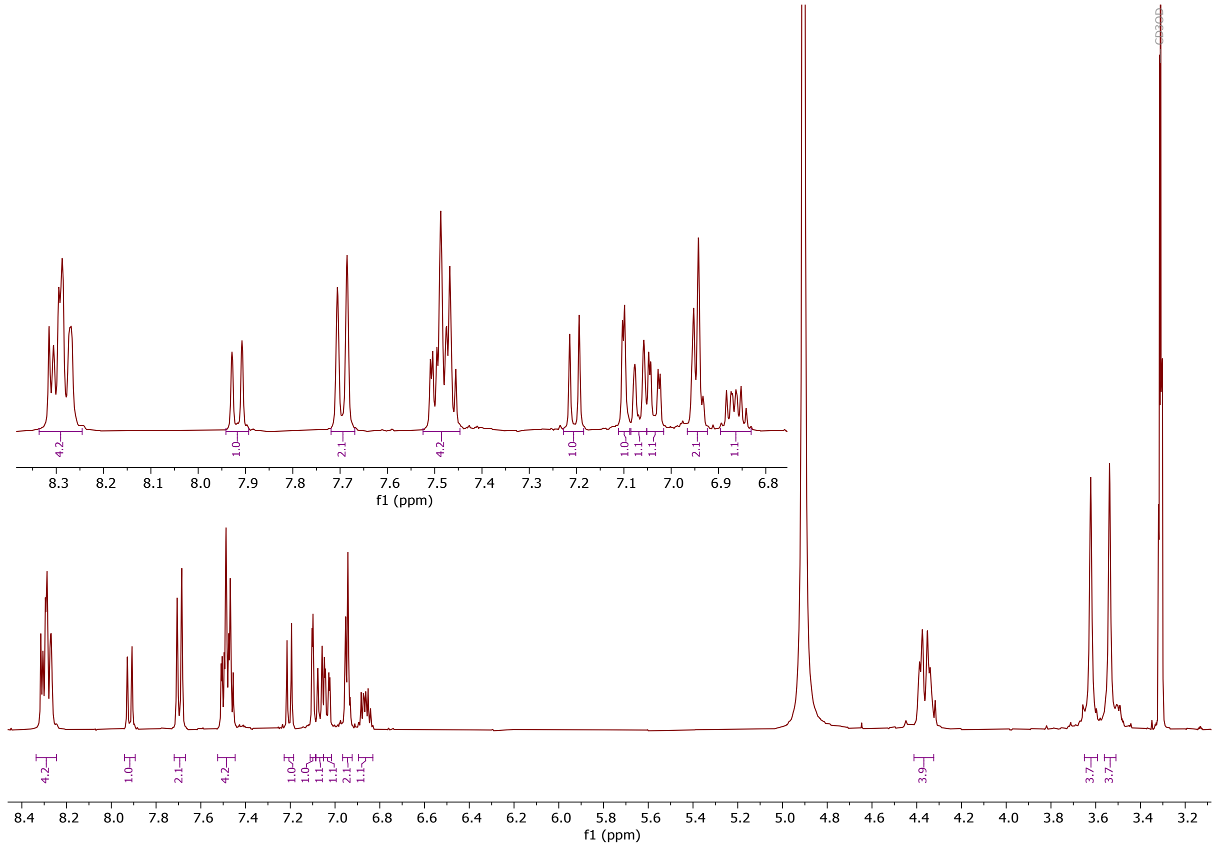

Figure S.2.6. ^{1}H – NMR of the sensor **1** in methanol-*d4*.

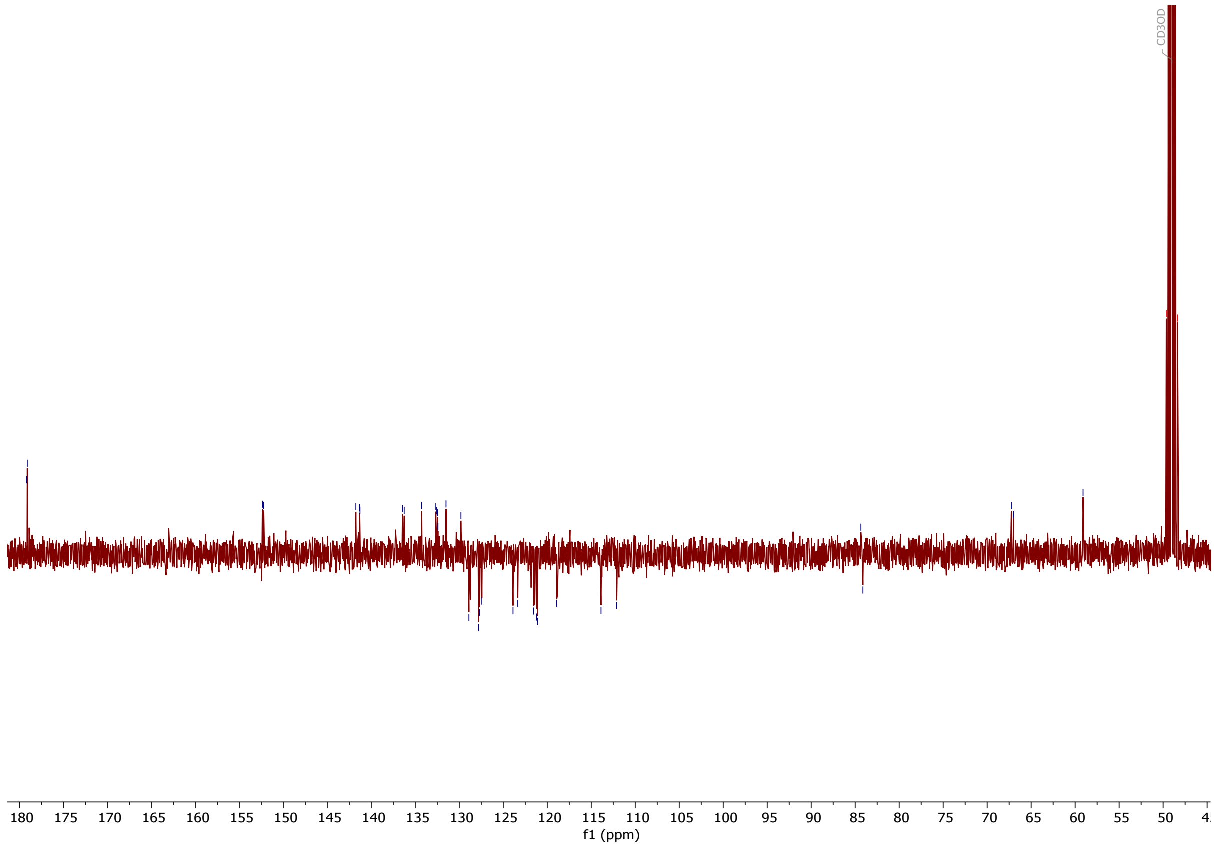

Figure S.2.7. ^{13}C – NMR of the sensor **1** in methanol-d_4.

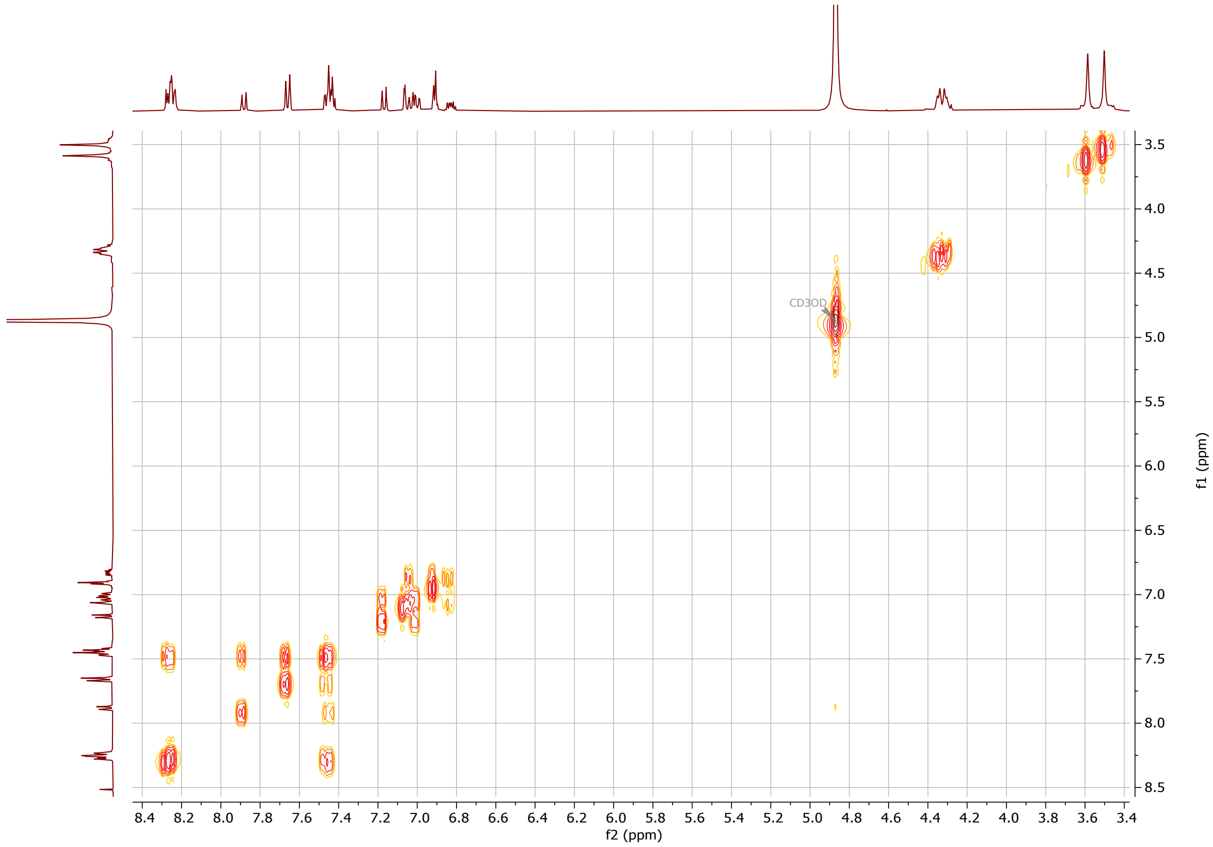

Figure S.2.8. ^{1}H – COSY NMR of the sensor **1** in methanol-d_4.

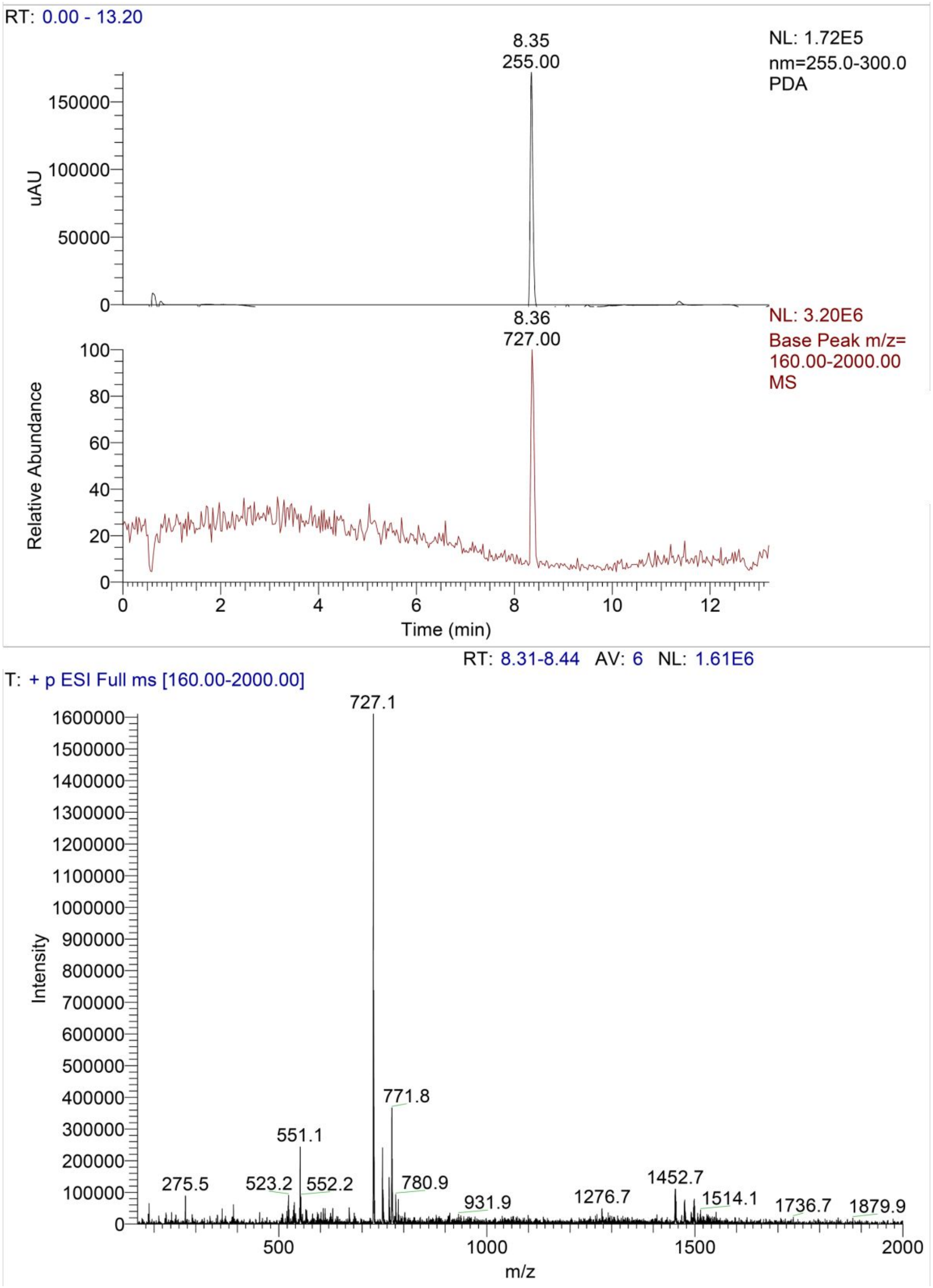

Figure S.2.9. HPLC data of the final compound **1**.

2. NMR spectroscopy

2.1. ^{1}H – NMR titration

^{1}H-NMR titration was performed with Brucker AV-400 at room temperature. For the first four experiments solutions of the sensor **1** in methanol-d_4 (3 mM) with different Ca^{2+} concentrations (0 mM; 0.75 mM; 1.5 mM; 3 mM) were used. A $CaCl_2x2H_2O$ solution in methanol-d_4 was used as a source of Ca^{2+}. For the last 3 experiments solutions of the sensor **1** in methanol-d_4 (1 mM) with different Ca^{2+} concentrations (1.5 mM; 2 mM; 1.5 mM; 2.5 mM) were prepared. After addition of more than 1:1 Ca^{2+} ratio a precipitate was observed.

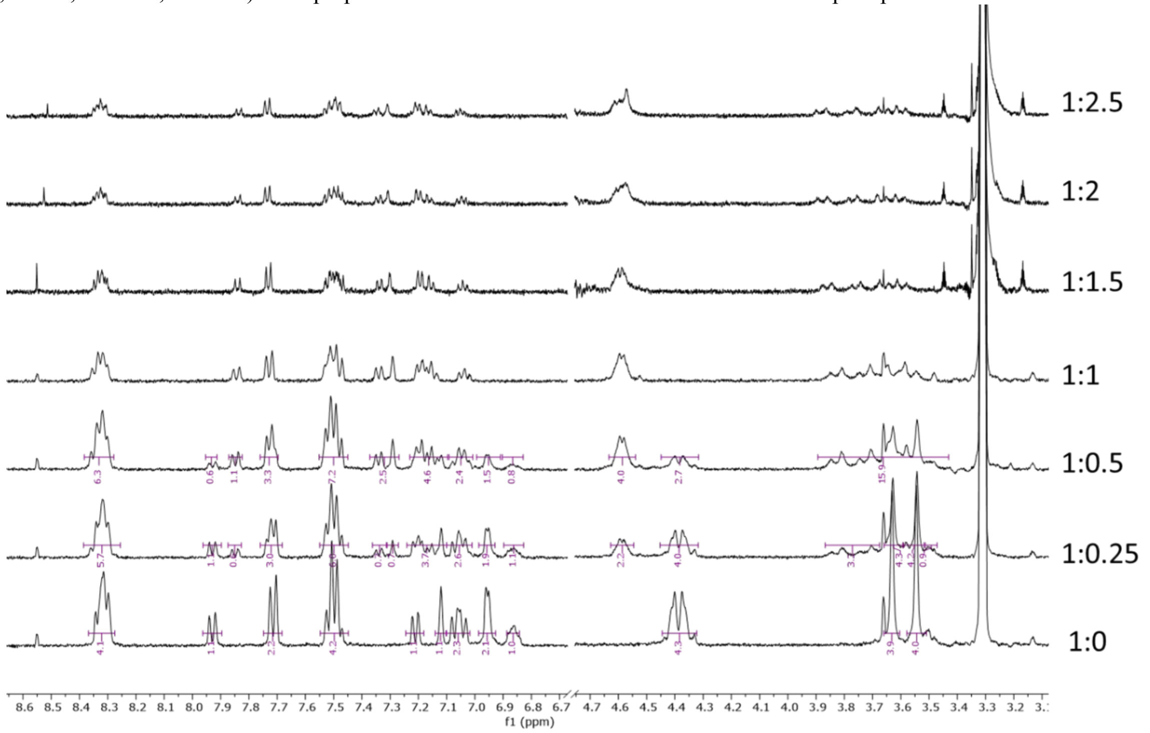

Figure S.3.1 ^{1}H-NMR titration data of sensor **1** solution in methanol-d_4 (1 mM) in presence of different Ca^{2+} concentrations. The sensor:Ca^{2+} ratios are indicated.

2.2. ^{1}H-DOSY spectroscopy

^{1}H-DOSY experiments were made with AV-III-600 at 298 K in 3 mm EPR tubes. Three samples with different sensor **1** – Ca^{2+} ratios were prepared as follows: in methanol-d_4 the sensor **1** (3 mM) without Ca^{2+}; with 1.5 mM Ca^{2+} and with 3 mM Ca^{2+}. $CaCl_2x2H_2O$ solution in methanol-d_4 was used as a source of Ca^{2+}. Diffusion delays d20 and p30 were selected in such a way that ratio between intensity of GPZ6 5% and GPZ6 95% peaks were 0.05. They are d20 = 90 ms and p30 = 480 µs. The obtained data were processed with Brucker TopSpin software. Diffusion coefficients were determined with the Stejskal-Tanner equation fitting of T1/T2 relaxation module analysis of integrated signals (Figure S.3.2). Diffusion coefficients were corrected with respect to equation S.3.1 by using water in methanol-d_4 diffusion coefficient as an internal reference.[2,3]

$$logD_{x,w.corr} = logD_{w.fix} - logD_w + logD_x$$ Equation S.3.1.

where $D_{x,w.corr}$ – corrected diffusion coefficient of the target protons, $D_{w.fix}$ – water in methanol-d4 diffusion coefficient at 298 K from literature, D_w – recorded water in methanol-d4 diffusion coefficient, D_x – recorded diffusion coefficient of the target protons.
Hydrodynamic radii (**r_H**) were calculated using the Stokes-Einstein equation (equation S.3.2, Table S.3.2, Figure S.3.3).

$$D = \frac{k_B T}{6\pi\eta r_H},$$ Equation S.3.2

where D - diffusion coefficient, k_B – Boltzmann constant, T – temperature in Kelvin and r_H – hydrodynamic radius

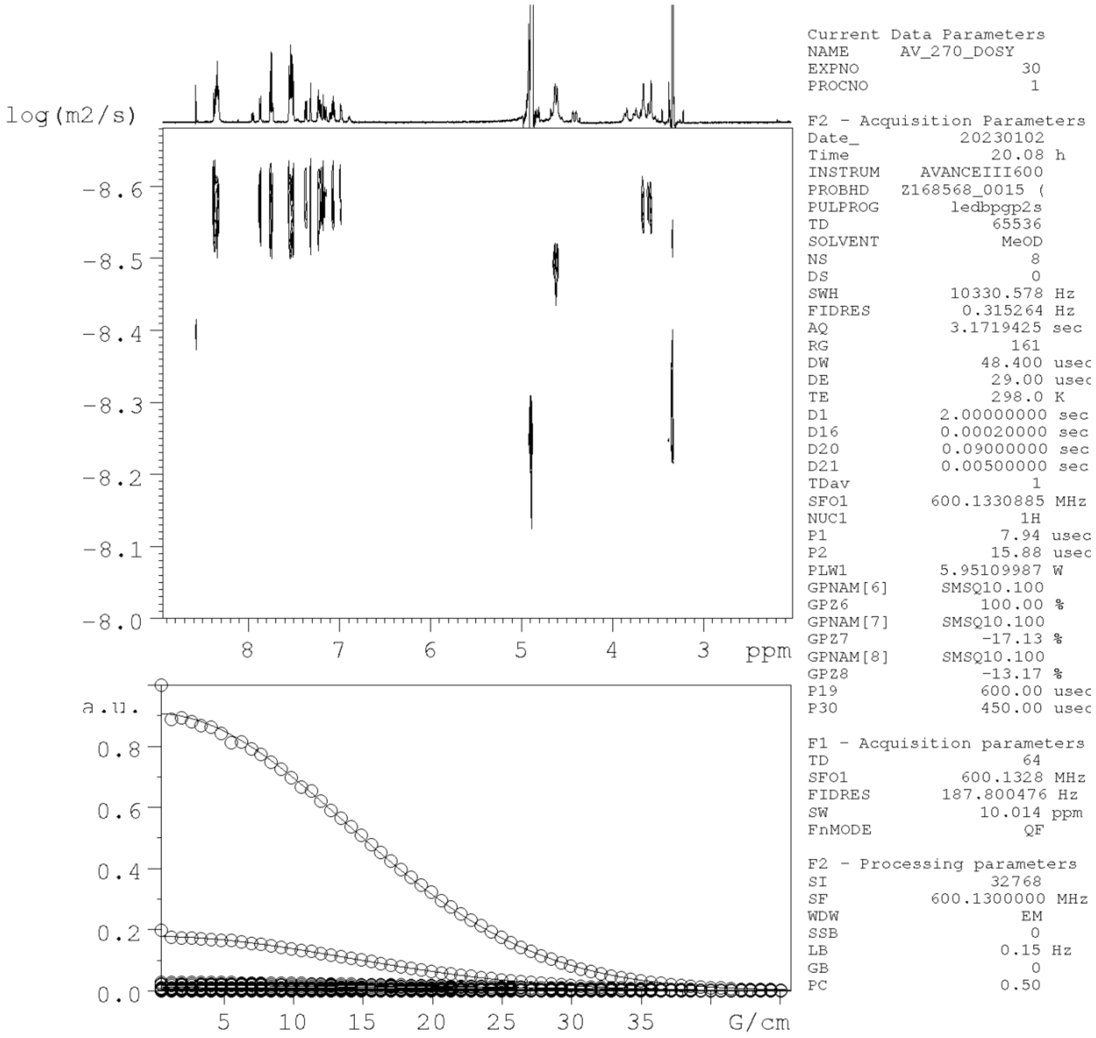

Figure S.3.2. ^{1}H-DOSY spectrum (600 MHz, 298 K, methanol-d4) of sensor **1** (3 mM) and $CaCl_2x2H_2O$ (1.5 mM), corrected diffusion coefficient = 7.4639x10^{-10} m^2/s.; T1/T2 analysis and acquisition parameters are indicated on the right.

Table S.3.2. Diffusion coefficient values (D) and calculated hydrodynamic radius (r_H) for sensor 1 in presence of different Ca^{2+} concentrations.

Sensor 1 conentration, mM	Ca^{2+} concentration, mM	D_x (m^2s^{-1})	D_w(m^2s^{-1})	$D_{x,w.corr}$ (m^2s^{-1})[a]	log $D_{x,w.corr}$	r_H (Å)
3	0.0	2.67x10^{-9}	5.44x10^{-9}	9.536x10^{-10}	-8.5530	4.2
3	1.5	1.7610^{-9}	5.11x10^{-9}	7.906x10^{-10}	-8.6932	5.12
3	3.0	1.91x10^{-9}	4.97 x10^{-9}	7.464x10^{-10}	-8.7241	5.4

[a] $D_{w.fix}$ = 1.94x10^{-9}.[3]

$$D = \frac{k_B T}{6\pi\eta r_H},$$ Equation S.3.2

where D - diffusion coefficient, k_B – Boltzmann constant, T – temperature and r_H – hydrodynamic radius

.

3. Isothermal calorimetry titration experiments.

3.1. In water solution

Binding constants were determined with MicroCal VP-ITC. In HEPES buffer (10 mM, pH = 7.2) two solutions of sensor **1** (100 μM) as a titrated sample and $CaCl_2x2H_2O$ (1 mM) as an titrating solution were prepared. The samples were degassed in MicroCal ThermoVac during 5 min under vacuum at 19 ^oC prior to measurements. Titration was performed at 20 ^oC with 28 injections (2 μL first followed by 10 μL). Milli-Q water was used as a reference, reference power was set for 10 μcal/s, initial delay 60 s, stirring speed 307 rpm. The obtained data were processed with MicroCal ITC-ORIGIN Analysis Software (Figure S.4.1). Last injection of 10 uL was deleted. Data were fitted with two sites mode. Experiment was repeated three times. As a result of three experiments estimated binding constants are: K_1 = 2.64 x10^6±1876 M^{-1} (ΔH_1 = 626.6±12.3 cal/mol; ΔS_1 = 31.4 cal/mol/deg); K_2 = 5.05x10^4±135 M^{-1} (ΔH_2 = -1.03x10^4±60 cal/mol; ΔS_2 = -13.6 cal/mol/deg) (Table S.4.1., Equation S.4.1).

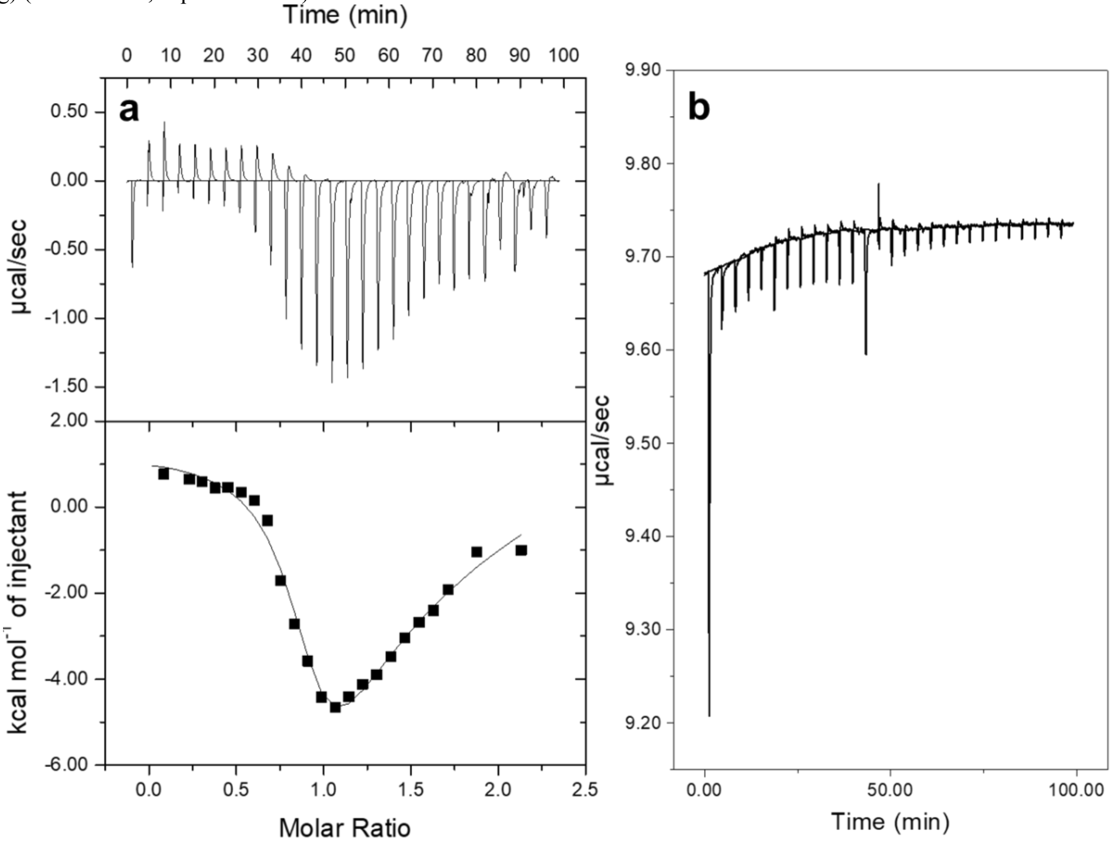

Figure S.4.1. Isothermal calorimetry data of sensor **1** titration with $CaCl_2$•$2H_2O$ in 10 mM HEPES buffer at 20 ^oC. a) ITC data for titration; b) reference data for buffer titration with the same Ca^{2+} solution.

Table S.4.1. Binding constants and error calculation

n/n	N_1, sites	K_1, M^{-1}	ΔH_1, kJ/mol	ΔS_1, J/mol/K	N_2, sites	K_2, M^{-1}	ΔH_2, kJ/mol	ΔS_2, J/mol/K
1	0.795±0.0142	1.76x10^6±3.82x10^5	1.5±0.2	124	0.457±0.063	4.49x10^4±6.23	-51.1±8.4	-0.09
2	0.761±0.0133	2.21x10^6±5.78x10^5	2.3±80.3	129	0.529±0.063	4.88x10^4±7.46	-42.3±6.3	-0.05
3	0.746±0.0161	3.94x10^6±1.84x10^6	4.0±0.5	140	0.611±0.085	5.78x10^4±1.74x10^4	-35.6±6.9	-0.03
average±s[a]	0.767±0.058	2.64x10^6±1876	2.62±0.05	131	0.523±0.334	5.05x10^4±135	-43±0.3	-0.06

a. calculated from equation S.4.1.

$$\left(\frac{\sigma}{A}\right)^2 = \sum_{n=1}^{n} \left(\frac{\sigma_n}{N}\right)^2, \qquad \text{Equation S.4.1.}$$

Where A is the average of the measured parameter, s is the error, s_n is the error of the n measurement, and N is the measured parameter value.

3.2. In methanol

In methanol two solutions of the sensor **1** (100 µM) as a titrated sample and $CaCl_2 \cdot 2H_2O$ (1 mM) as titrating solution were prepared. The samples were degassed in MicroCal ThermoVac during 3 minutes under vacuum at 19 °C prior to measurements. Titration was performed at 20 °C with 28 injections (2 µL first followed by 10 µL). Methanol was used as a reference, reference power was set for 10 µcal/s, initial delay 60 s, stirring speed 307 rpm. The obtained data were processed with MicroCal ITC-ORIGIN Analysis Software. In figure S.4.2 we observe several processes upon increasing the calcium concentration. We assume that from 0.0 to 0.5 molar guest:host ratio binding is clearly an entropically-driven process assigned to binding to calcium of 1 to 1 ratio. After 1:1 host:guest ratio two enthalpy-driven processes seem to occur.

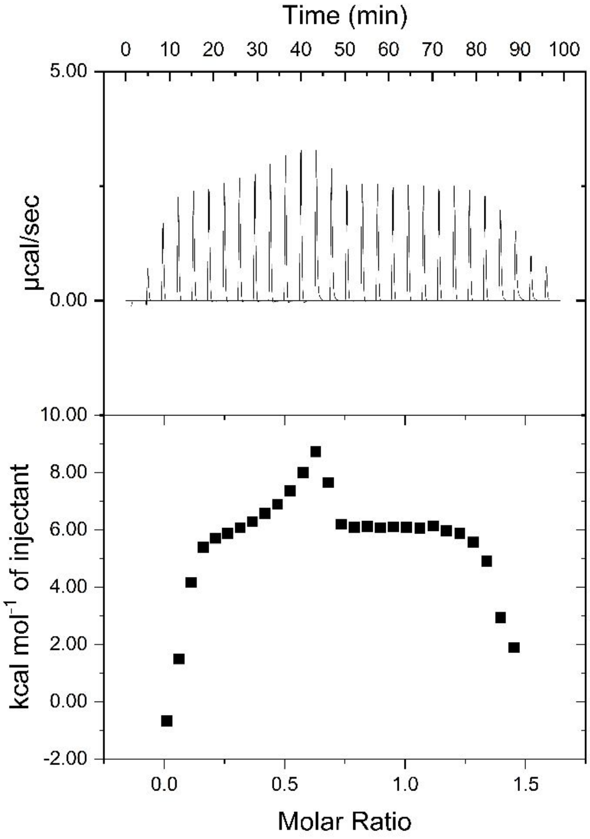

Figure S.4.2. Isothermal calorimetry data of sensor **1** titration with calcium in methanol at 20 °C.

4. Steady-state spectroscopy

4.1. Fluorescence titration

Fluorescence emission spectra were recorded with HORIBA Aqualog spectrometer in a 1 cm quarz cuvette at 21 °C. Sensor **1** (6 µM) was titrated with $CaCl_2x2H_2O$ (0.05 mM, 0.5 mM, 5 mM, 20 mM and 100 mM). All solutions were prepared in methanol. Recorded spectra were processed with Origin 2022 software.

4.2. Fluorescence quantum yield measurements

Fluorescence quantum yield (QY) was estimated by comparison with perylene fluorescence QY in cyclohexene (0.94),[4] and calculated by equation S.5.1.:

$$\Phi_f = \Phi_{fR}\frac{F\ \mathrm{OD}_R n^2}{F_R\ \mathrm{OD}\ n_R^2}, \qquad \text{Equation S.5.1.}$$

Where Φ_f is a fluorescence QY of the molecule of interest; Φ_{fR} is a fluorescence QY of the reference; F is the integrated fluorescence intensity, OD is the optical density, and n is the refractive index of the solvent.
Solutions of the sensor **1** in methanol and perylene in cyclohexane were prepared in such a concentration that its OD at 400 nm wavelength was below 0.05. Emission spectra were recorded with HORIBA Aqualog spectrometer in a 1 cm quarz cuvette at 21 °C. Recorded spectra were processed with Origin 2022 software.

Table S.5.1. Fluorescence quantum yields for 1 in presence of different Ca^{2+} concentrations calculated with equation S.5.1.

1, [µM]	Ca^{2+}, [µM]	OD	n	QY
5	0	0.056	1.33	0.18
5	0.001	0.057	1.33	0.19
5	0.01	0.057	1.33	0.19
5	0.05	0.057	1.33	0.19
5	0.1	0.057	1.33	0.20
5	0.5	0.058	1.33	0.25
5	1	0.058	1.33	0.29
5	1.5	0.058	1.33	0.32
5	3	0.058	1.33	0.35
5	5	0.058	1.33	0.38
5	7	0.058	1.33	0.39
5	9	0.058	1.33	0.39
5	10	0.057	1.33	0.39
5	20	0.058	1.33	0.39
5	40	0.058	1.33	0.40
5	80	0.057	1.33	0.42
5	160	0.058	1.33	0.43
5	320	0.058	1.33	0.45
5	640	0.057	1.33	0.45
5	1280	0.057	1.33	0.45
Perylene[a]	-	0.026	1.44	0.94

[a] Reference sample in cyclohexane with QY 0.94.[4]

4.3. Selectivity and competition studies

Selectivity study.

Solutions of sensor **1** (4 µM), NaOAc (300 mM), KOAc (1 M), $MgCl_2$ (10 mM), $MnSO_4 \cdot H_2O$, $FeSO_4 \cdot 7H_2O$, $CoSO_4 \cdot 7H_2O$, $NiSO_4 \cdot 6H_2O$, $CuSO_4 \cdot 5H_2O$, $ZnSO_4 \cdot 7H_2O$ (1 mM) and $CaCl_2 \cdot 2H_2O$ (1 mM) were prepared in methanol. The sensor solutions were transferred to 1x1 fluorescence quartz cuvette. Fluorescence measurements were performed with methanol as a blank at 21°C, λ_{ex} = 400 nm. After measurement of the metal free solutions, the necessary amount of metal cation stock solution was added: NaOAc (0.15 mL), KOAc (0.42 mL), $MgCl_2$ (0.15 mL), $MnSO_4 \cdot H_2O$, $FeSO_4 \cdot 7H_2O$, $CoSO_4 \cdot 7H_2O$, $NiSO_4 \cdot 6H_2O$, $CuSO_4 \cdot 5H_2O$, $ZnSO_4 \cdot 7H_2O$ (10 µL) and $CaCl_2 \cdot 2H_2O$ (3 µL), followed by the measurements repeating Emission spectra were recorded with HORIBA Aqualog spectrometer in a 1 cm quarz cuvette at 21 °C. Recorded spectra were processed with Origin 2022 software.

Competition study.

Solutions of NaOAc (15 mM), KOAc (140 mM), $MgCl_2$ (0.5 mM), $MnSO_4 \cdot H_2O$, $FeSO_4 \cdot 7H_2O$, $CoSO_4 \cdot 7H_2O$, $NiSO_4 \cdot 6H_2O$, $CuSO_4 \cdot 5H_2O$, $ZnSO_4 \cdot 7H_2O$ (1 µM) with sensor **1** (6 µM) each were prepared in methanol. $CaCl_2 \cdot 2H_2O$ (1 mM) stock solution was prepared separately. Obtained solutions of the sensor and metal salts were transferred to 1x1 cm fluorescence quarz cuvettes. Fluorescence measurements were performed with methanol as a blank at 21°C, λ_{ex} = 400 nm. After measurement of the calcium free solutions without, 3 µL of $CaCl_2 \cdot 2H_2O$ stock solution (1 µM) had been added and the measurements repeated. Emission spectra were recorded with HORIBA Aqualog spectrometer in a 1 cm quarz cuvette at 21 °C. Recorded spectra were processed with Origin 2022 software (Figure S.5.1).

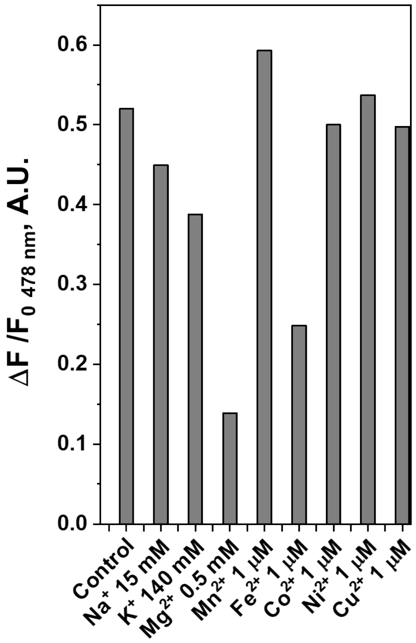

Figure S.5.1. Competition study: Solutions of metal cations with 6 µM of **1** were prepared in methanol, after which Ca^{2+} (1 µM) was added, samples were irradiated with 400 nm light. Control is the $\Delta F/F_0$ of the sensor in the presence of 1 µM of Ca^{2+}.

4.4. Upconversion emission spectroscopy

4.4.1. Upconversion power dependence measurements.

Power dependence spectra were recorded with a custom-build setup (figure S.5.2 a) reported previously.[5] The excitation power was measured with a Thorlabs® PM100USB power meter. Power density was obtained by dividing of the light power by the laser beam cross-section. For power dependence measurement a sample containing Pd-TPTBP (2.5 µM), the sensor **1** (75 µM) and $CaCl_2x2H_2O$ (75 µM) in methanol was freshly prepared in 0.25 x 1 cm (optical path 1 cm) fluorescence cuvette. The cuvette was placed in the cuvette holder **5** in such a way that the short side was facing the collimator **3**. The sample was degassed by purging argon during 30 min prior to measurement. Spectra were recorded with AvaSoft® software and processed with Origin 2022®.

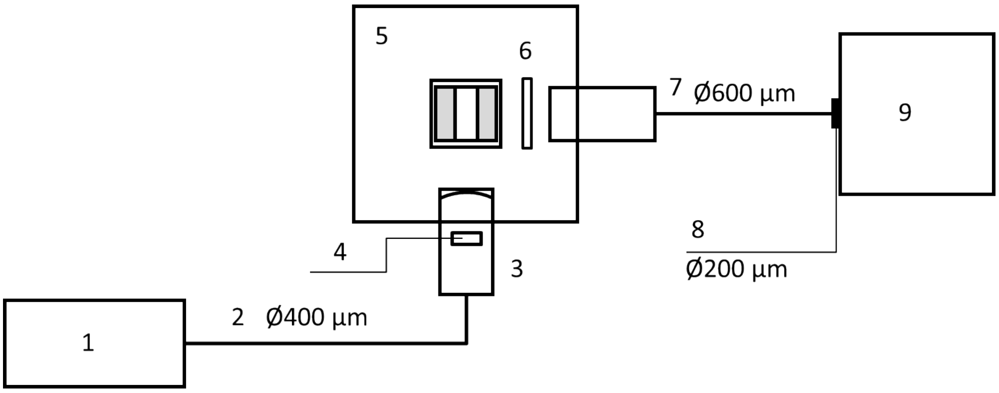

Figure S.5.2. Setup for upconversion emission spectroscopy. 1) laser source for 635 nm; 2) fibre-optic cable FC-UVIR400-2 from Avantes; 3) combined together collimator and bandpass filter 4) Bandpass filter FLH05635-10 form Thorlabs (CWL = 635 nm, FWHM = 10 nm); 5) cuvette holder with temperature control; 6) notch filter NF633-25 from Thorlabs (633 nm, 50% band width = 25 nm); 7) optical fiber FC-UVIR600-2 from Avantes®; 8) slit with diameter 200 µm from Avantes®; 9) StarLine AvaSpec-2048L spectrometer from Avantes®.

4.4.2. Phosphorescence quenching in presence of the annihilator 1

Steady-state emission spectra were recorded with the same setup described above. 635 nm laser (16.2 mW, 2.9 mm^2, 559 mW/cm^2) was used as an irradiation source. All measurements were made at 24 °C temperature. In in 0.25 x 1 cm (optical path 1 cm) quarz cuvette equipped with a septum a solution of PdTPTBP (2.5 µM) in methanol was prepared and deoxygenated by means of purging with argon during 20 min. After addition of a portion of the annihilator **1** solution the sample was further deoxygenated during 10 min. The recorded spectra were processed with AvaSoft® and OriginPro 2022® software (Figure S.5.3).

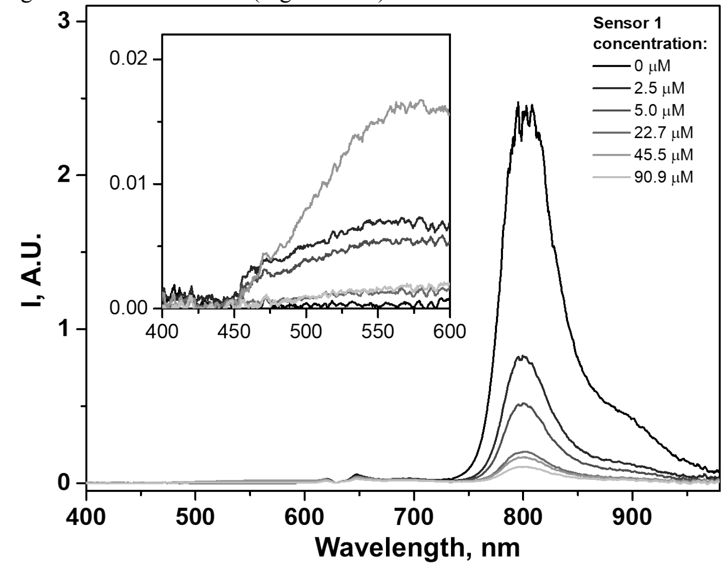

Figure S.5.3. Pd-TPTBP phosphorescence quenching in presence of different concentrations of annihilator **1**. Conditions: λ_{ex} = 635 nm, laser power = 16.2 mW, laser beam cross-section area = 2.9 mm^2; power density = 559 mW/cm^2, methanol; 24 °C. Insert shows a zoom of the 400-600 nm emission region.

4.4.3. Titration of the PS and the sensor 1 solution in methanol in deoxygenated conditions with Ca^{2+} at r.t.

Emission spectra were recorded with the setup described above. 635 nm laser (16.2 mW, 2.9 mm^2, 559 mW/cm^2) was used as an irradiation source. All measurements were made at 24 °C temperature. In in 0.25 x 1 cm (optical path 0.675 cm) quarz cuvette equipped with a septum a solution of PdTPTBP (2.5 µM) and the sensor **1** (75 µM) in methanol was prepared and deoxygenated by means of purging with argon during 20 min. Separately, a solution of $CaCl_2$•$2H_2O$ (1 mM) was prepared in methanol. After addition of a portion of the $CaCl_2$•$2H_2O$ solution, the sample was further deoxygenated during 10 min. The recorded spectra were proceeded with AvaSoft® and OriginPro 2022® software.

4.4.4. Control titration of the PS and perylene solution in methanol in deoxygenated conditions with Ca^{2+} at r.t.

Emission spectra were recorded with the setup described above. 635 nm laser (16.2 mW, 2.9 mm^2, 559 mW/cm^2) was used as an irradiation source. All measurements were made at 24 °C temperature. In in 0.25 x 1 cm (optical path 0.675 cm) quarz cuvette equipped with a septum a solution of PdTPTBP (2.5 μM) and perylene (75 μM) in methanol was prepared and deoxygenated by means of purging with argon during 20 min. Separately, a solution of $CaCl_2$•$2H_2O$ (1 mM) was prepared in methanol. After addition of a portion of the $CaCl_2$•$2H_2O$ solution, the sample was further deoxygenated during 10 min. The recorded spectra were proceeded with AvaSoft® and OriginPro 2022® software (Figure S.5.4).

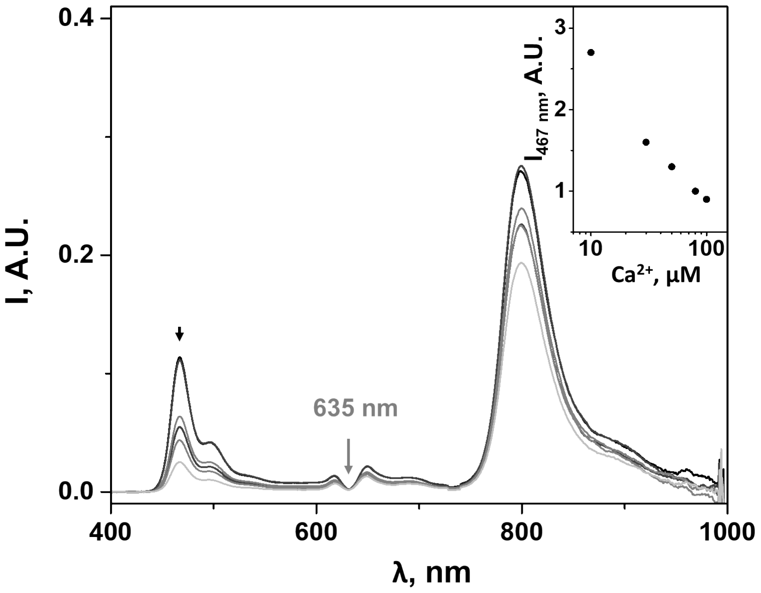

Figure S.5.4. Titration of PdTPTBP (2.5 μM) and perylene (75 μM) solution in methanol in deoxygenated conditions with Ca^{2+}. Conditions: λ_{ex} = 635 nm, laser power = 16.2 mW, laser beam cross-section area = 2.9 mm^2; power density = 559 mW/cm^2, methanol; 24 °C.

4.5. Quantum yield measurements by absolute method

The measurement was performed with a custom-build setup (Figure S.5.5).

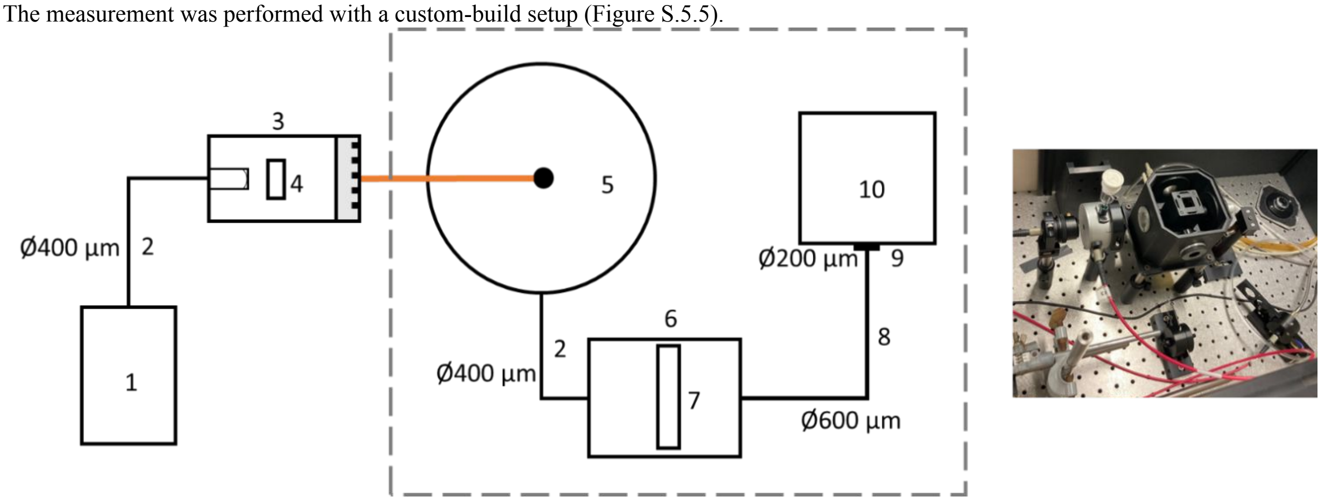

Figure S.5.5. Setup for quantum yield measurements by absolute method. 1) laser source (635 nm); 2) optical fiber FC-UVIR400-2 from Avantes; 3) combined collimator F220SMA-B from Thorlabs (= 633 nm, f = 10.99 mm, NA = 0.25), bandpass filter and mechanical iris; 4) Bandpass filter FLH05635-10 form Thorlabs (CWL = 635 nm, FWHM = 10 nm); 5) modified integrating sphere from Avantes with a sample holder; 6) filter holder; 7) neutral density filter OD 2.0 from Edmund or 565 nm short pass filter, or notch filter NF633-25 from Thorlabs (633 nm, 50% band width = 25 nm); 8) optical fiber FC-UVIR600-2 from Avantes®; 9) slit with diameter 200 µm from Avantes®; 10) StarLine AvaSpec-2048L spectrometer from Avantes®. Part of the setup dedicated with dash line was calibrated by means of Avalight-HAL-CAL-ISP30 NIST traceable calibration lamp from Avantes® with published procedure https://www.youtube.com/watch?v=Qbn-DIabp0E, filter holder (6) was empty during the calibration.

The procedure for quantum yield determination had been adapted from the work previously made in our group. [6] The luminescence spectrum of a blank sample with pure methanol was measured first. For that sample, an OD 2.0 neutral density filter was placed in the filter holder **6** to dim the laser light and protect the sensor. Then a sample containing Pd-TPTBP (2.5 μM), sensor **1** (25 μM or 75 μM) and $CaCl_2x2H_2O$ (75 μM) in methanol was degassed by purging argon during 30 min. A luminescence spectrum of this sample was then recorded. The neutral

density filter was replaced by a 625 nm short-pass filter, and the upconverted luminescence spectra was recorded. The obtained data were processed with OriginPro 2022® software (Figure S.5.6) using the equation defining the upconversion quantum yield (Equation S.5.2):

$$\Phi_{UC} = \frac{\int_{\lambda_1}^{\lambda_2}\left(\frac{I_{annihilator}(\lambda)}{T(\lambda)}\right)d\lambda}{\int_{\lambda_3}^{\lambda_4}\left(\frac{I_{exc-blank}(\lambda)-I_{exc-sample}(\lambda)}{OD2\ transmittance}\right)d\lambda} \equiv \frac{q_{p-em}}{q_{p-abs}}\ , \qquad \text{Equation S.5.2.}$$

where λ_1-λ_2 - wavelengths range of upconverted signal; λ_3-λ_4 – wavelengths range of laser signal luminescence for the blank and the TTA-UC samples, $T_{SP625}(\lambda)$ – transmittance of the 625 nm short pass filter, $T_{OD2.0}(\lambda)$ – transmittance of the neutral density filter with OD = 2.0.

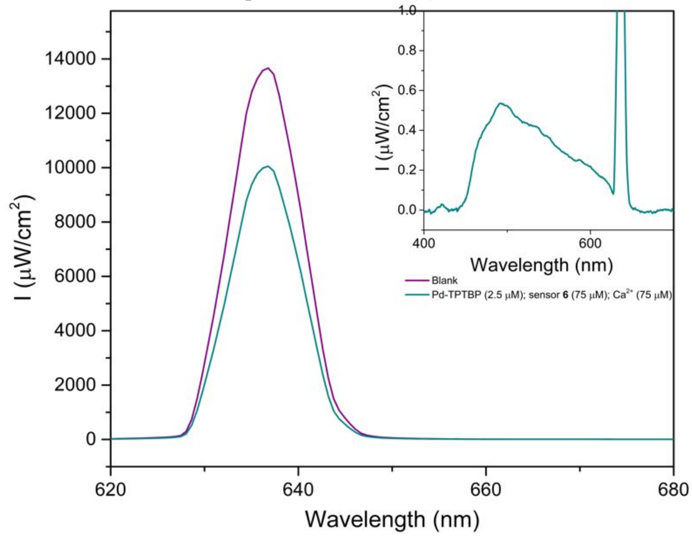

Figure S.5.6. Laser signal of the blank and TTA-UC samples and its upconverted emission. Conditions: λ_{ex} = 635 nm, laser power = 16.2 mW, laser beam cross-section area = 2.9 mm^2; power density = 559 mW/cm^2; solvent: methanol; temperature: 24 ^{o}C., argon.

The recorded upconverted emission spectra and laser signal for the blank sample and the TTA-UC sample were corrected with transmission of 625 nm short-pass and OD 2.0 neutral density filters, respectively. Obtained spectra were integrated in the ranges λ_1-λ_2 = 440 nm - 625 nm and λ_3-λ_4 = 625 nm - 649 nm. The final upconversion quantum yield was calculated according to Equation S.5.3:

$$\Phi_{UC} \equiv \frac{q_{p-em}}{q_{p-abs}} = \frac{99}{47922} = 0.002, \qquad \text{Equation S.5.3}$$

Where q_{p-em} is the emitted photon flux of the upconverted luminescence [photons/s] and q_{p-abs} is the absorbed photon flux by the photosensitizer [photons/s].

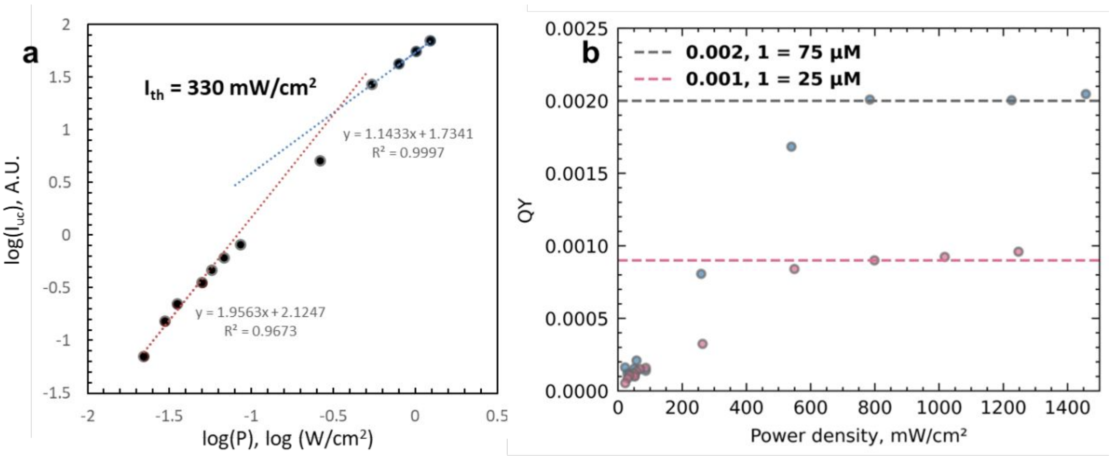

Figure S.5.7. I_{th} of PS (2.5 μM) and **1** (25 μM) system determination in the presence of 75 μM of Ca^{2+} in methanol. b) Quantum yield dependence of power density for PS (2.5 μM) and **1** (25 μM and 75 μM). Conditions: λ_{ex} = 635 nm laser beam cross-section area = 5.7 mm^2; solvent: methanol; temperature: 24 ^{o}C, argon.

4.6. Beam profiling

A combination of CinCam CMOS-1201-Nano with neutral density filter OD 2.0 from Edmund, placed between the camera and the laser beam source was used for cross section area measurement. Beam diameters and cross section area were determined with RayCiLite software. The 10% laser beam diameter were determined by Gauss Distribution fitting in RayCiLite software (Figure S.5.6). The obtained cross section area in mm^2 was used for power density calculation in parts 5.4 and 5.5.

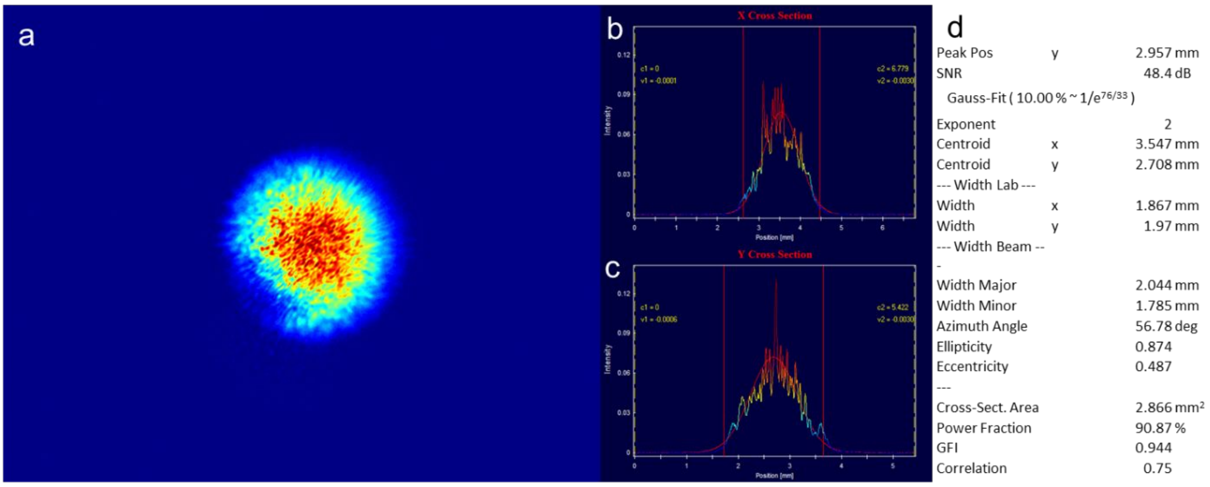

Figure S.5.1. Example of beam profiling data obtained with RayCiLite. a) 2D cross-section of laser beam; b) X Cross section of the beam; c) Y cross section of the beam; d) result of fitting performed in the software.

5. Time-resolved spectroscopy

5.1. Fluorescence lifetime measurements

A combination of a Tsunami® Ultrafast Ti:Sapphire Osscilator, Hamamatsu® HPDTA streak camera C4334, a Newport Spectra-Physics® pulse selector (model 3980) and a CHROMEX spectrograph was used for recording of fluorescence decay spectra [7]. Ones were recorded in deaerated and aerated conditions in methanol with the 420 - 565 nm spectral range and 10 ns time window. Pulses centered at 400 nm were used for sample excitation. Instrumental response factor was recorded with a glass reflecting the excitation beam into the detector. Obtained spectra processed with Origin 2022® software. Samples with the sensor **1** (6 μM) and different Ca^{2+} concentrations (0 μM; 0.5 μM; 2.5 μM; 1 μM; 7 μM and 10 μM) were freshly prepared in deoxygenated conditions in 1x1 cm fluorescence quartz cuvettes equipped with Schlenk line connectors with valves. For recording spectra in aerated conditions, the valves were left open for 20 min and the samples were used the second time.

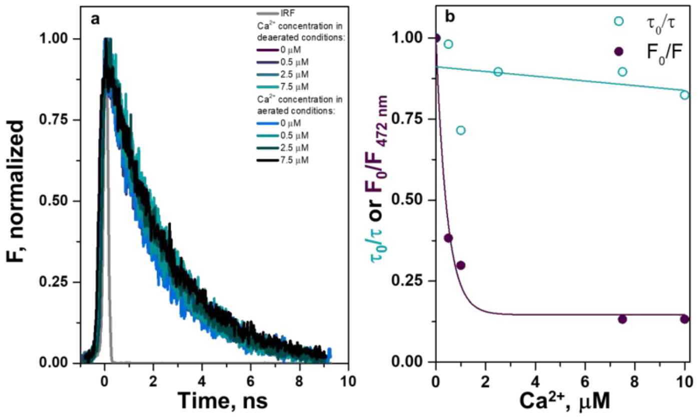

Figure S.6.1. Normalized fluorescence decay of sensor **1** in presence of different Ca^{2+} concentrations (a) and the plot of the ratio of singlet state lifetimes of sensor **1** and it's fluorescence intensities ratio versus Ca^{2+} concentration (b). Here we observe a static interaction of sensor **1** with Ca^{2+}.

5.2. Nanosecond transient absorption spectroscopy

Transient absorption spectra were recorded with a custom-built setup described previously [8]. Samples were excited with 627 nm laser pump (The Continuum® Surelite™ Nd:YAG laser in combination with The Continuum® OPO Plus) with the power of 200 μJ. A 75 W Xenon arc lamp was used as a probe. 610 nm short-pass or 640 nm long-pass filters were placed before the detector to avoid the pump-scattering. Measurements were made in 1 cm fluorescence quartz cuvettes modified with a Schlenk connecting adapter. Stability of the samples were checked before and after the measurement with recording of their Uv-vis spectra. Recorded spectra were processed with Origin 2022® software. For Stern-Volmer plot, different samples containing Pd-TPTBP (5 μM) and different sensor **1** concentrations (0 μM; 25 μM; 75 μM and 125 μM) in deaerated methanol were freshly prepared. The spectra were recorded in the range 350 – 800 nm. For the Stern-Volmer plot lifetimes t_l were taken (Figure S.6.2, 4b).

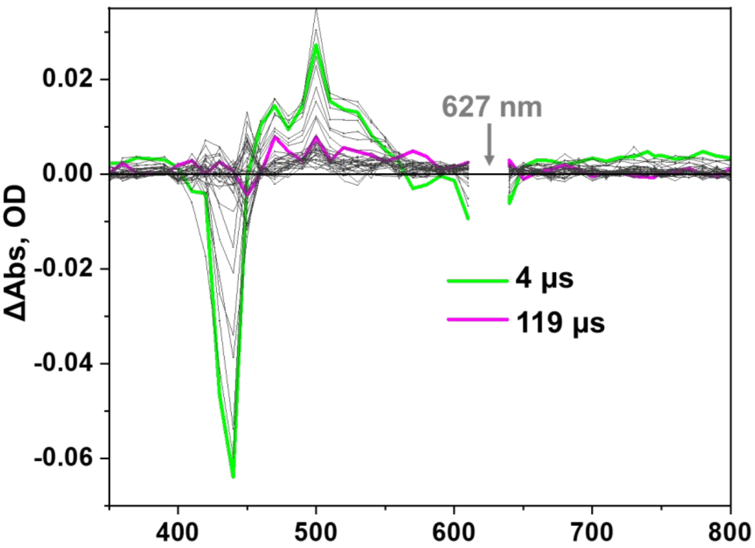

Figure S.6.2. Transient absorption spectrum of PdTPTBP (5 µM) and **1** (75 µM) sample in methanol in deaerated conditions. The spectrum was fitted globally by biexponential decay function, revealing 4 µs (τ_1) and 119 µs (τ_2) lifetimes (λ_{pump} = 627 nm, 200 µJ).

For Ca^{2+}, different samples containing Pd-TPTBP (5 µM), sensor **1** (75 µM) and different concentrations of $CaCl_2 \times 2H_2O$ (0.6 µM, 1 µM, 10 µM and 100 µM) in deaerated methanol were freshly prepared. The spectra were recorded in the range 350 – 800 nm. The decays spectra at 500 nm were fitted with biexponential decay with Origin 2022® software (Figure S.6.3).

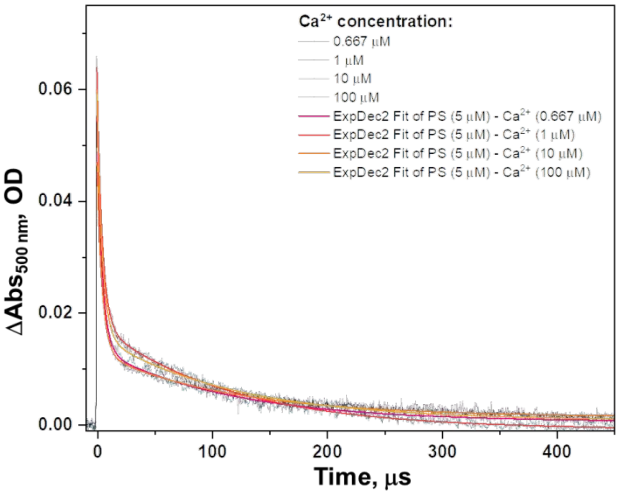

Model	ExpDec2			
Equation	y = A1*exp(-x/t1) + A2*exp(-x/t2) + y0			
Plot	\g(D)Abs/mOD (500 nm)	C	D	E
y0	5.67734E-4 ± 3.85706E-5	-7.86746E-4 ± 4.48498E-5	0.0015 ± 3.7149E-5	8.06787E-4 ± 4.0834E-5
A1	0.03302 ± 2.83874E-4	0.03671 ± 2.895E-4	0.02725 ± 2.82428E-4	0.03501 ± 2.8583E-4
t1*	4.49497 ± 0.04151	4.49497 ± 0.04151	4.49497 ± 0.04151	4.49497 ± 0.04151
A2	0.01296 ± 1.04484E-4	0.01871 ± 1.08592E-4	0.01133 ± 1.02579E-4	0.0152 ± 1.06097E-4
t2*	114.85881 ± 1.08324	114.85881 ± 1.08324	114.85881 ± 1.08324	114.85881 ± 1.08324
Reduced Chi-Sqr*	4.99642E-7			
R-Square (COD)	0.98463	0.99307	0.99027	0.97105
R-Square (COD)*	0.98485			
Adj. R-Square*	0.9848			

Figure S.6.3. Pd-TPTBP (5 µM) – Sensor **1** (75 µM) excited state absorption decay at 500 nm wavelength in presence of different Ca^{2+} concentrations. We don't observe change in biexponential decay lifetimes in presence of different calcium cation concentrations.

6. Computational studies

Geometry optimized models of sensor **1** with 0, 1, or 2 Ca^{2+} cations were constructed and optimized using ADF modeling suite from SCM.[9] Conformer searches were performed for each of the molecules using OpenBabel's genetic algorithm.[10] The resulting 20 conformers per sensor with 0, 1, and 2 Ca^{2+} ions bound were geometry optimized using TeraChem[11] using density functional theory (DFT: rev PBE0/6-311++g)[12] and COSMO to simulate solvation in water.

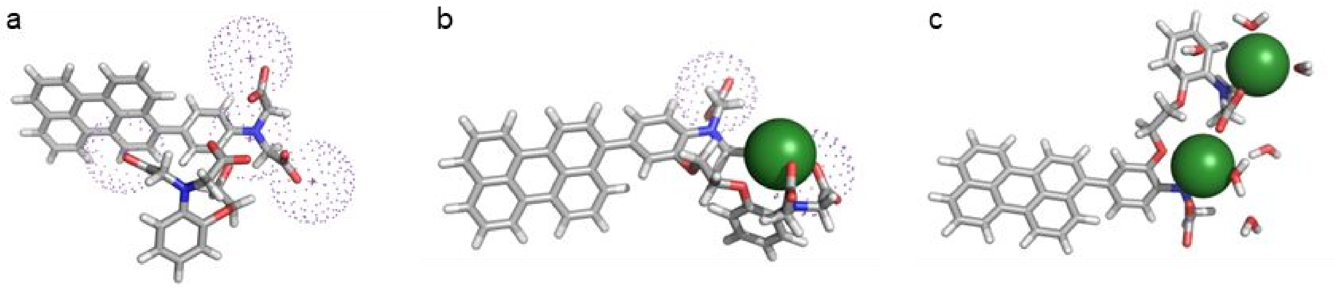

Figure S.7.1. DFT calculated models of a) [1·4Na]; b) [1·Ca·2Na]; c) [1·2Ca·6H_2O]. Na+ cations are represented as violet dotted spheres.

In order to evaluate the total energy of the two binding event and evaluate whether the formation of the 1:2 complex [**1**·2Ca·6H_2O] is entropically favorable, we considered the following 2-step balanced reaction. 4 sodium ions were added to keep the total charge of the system to zero. 12 water molecules were included in the model to stabilize the Ca^{2+} and Na^{+} ions and simulate their solvation when not bound or partially bound to sensor **1**. The multiplication coefficients are reported in table S.7.1.

$$1:0 \rightleftharpoons 1:1 \rightleftharpoons 1:2$$

$$2\,[Ca\cdot 6H_2O]^{2+} + 6\,H_2O + [\mathbf{1}\cdot 4Na] \rightleftharpoons [Ca\cdot 6H_2O]^{2+} + 2\,[Na\cdot 3H_2O]^{+} + 6\,H_2O + [\mathbf{1}\cdot Ca\cdot 2Na] \rightleftharpoons 4\,[Na\cdot 3H_2O]^{+} + [\mathbf{1}\cdot 2Ca\cdot 6H_2O]$$

Table S.7.1. DFT (revPBE0/6-311++g, COSMO) calculated energies of the components of the systems and multiplication coefficients to calculate the total energy of the systems. The ΔE values are referred to the previous state of the system.

Ratio **1**:Ca^{2+}	$[Ca\cdot 6H_2O]^{2+}$	$[Na\cdot 3H_2O]^{+}$	$6H_2O$	[**1**·4Na]	[**1**·Ca·2Na]	[**1**·2Ca·6H_2O]	E tot (kJ/mol)	ΔE (kJ/mol)
E (kJ/mol)	-2982231.72	-1027656.47	-1203699.36	-8214656.07	-9141530.20	-11272176.89	-	-
1:0	2	0	1	1	0	0	-15382818.88	0.00
1:1	1	2	1	0	1	0	-15382774.21	44.66
1:2	0	4	0	0	0	1	-15382802.75	-28.54

References

(1) Brown, K. E.; Veldkamp, B. S.; Co, D. T.; Wasielewski, M. R. Vibrational Dynamics of a Perylene–Perylenediimide Donor–Acceptor Dyad Probed with Femtosecond Stimulated Raman Spectroscopy. *J. Phys. Chem. Lett.* **2012**, *3* (17), 2362–2366. https://doi.org/10.1021/jz301107c.

(2) Neufeld, R.; Stalke, D. Accurate Molecular Weight Determination of Small Molecules via DOSY-NMR by Using External Calibration Curves with Normalized Diffusion Coefficients. *Chem. Sci.* **2015**, *6* (6), 3354–3364. https://doi.org/10.1039/C5SC00670H.

(3) Easteal, A. J.; Woolf, L. A. Diffusion of Water in Solutions of Alkali Metal Bromides, Tetraalkylammonium Bromides and Ammonium Bromide. *J Solution Chem* **1986**, *15* (12), 1003–1013. https://doi.org/10.1007/BF00645195.

(4) Berlman, I. B. *Handbook of Fluorescence Spectra of Aromatic Molecules*, 2d ed.; Academic Press: New York, 1971.

(5) Askes, S. H. C.; Pomp, W.; Hopkins, S. L.; Kros, A.; Wu, S.; Schmidt, T.; Bonnet, S. Imaging Upconverting Polymersomes in Cancer Cells: Biocompatible Antioxidants Brighten Triplet-Triplet Annihilation Upconversion. *Small* **2016**, *12* (40), 5579–5590. https://doi.org/10.1002/smll.201601708.

(6) Askes, S. H. C.; Bahreman, A.; Bonnet, S. Activation of a Photodissociative Ruthenium Complex by Triplet-Triplet Annihilation Upconversion in Liposomes. *Angew. Chem. Int. Ed.* **2014**, *53* (4), 1029–1033. https://doi.org/10.1002/anie.201309389.

(7) Portwich, F. L.; Carstensen, Y.; Dasgupta, A.; Kupfer, S.; Wyrwa, R.; Görls, H.; Eggeling, C.; Dietzek, B.; Gräfe, S.; Wächtler, M.; Kretschmer, R. A Highly Fluorescent Dinuclear Aluminium Complex with Near-Unity Quantum Yield. *Angew Chem Int Ed* **2022**, *61* (17), e202117499. https://doi.org/10.1002/anie.202117499.

(8) Amthor, S.; Knoll, S.; Heiland, M.; Zedler, L.; Li, C.; Nauroozi, D.; Tobaschus, W.; Mengele, A. K.; Anjass, M.; Schubert, U. S.; Dietzek-Ivanšić, B.; Rau, S.; Streb, C. A Photosensitizer–Polyoxometalate Dyad That Enables the Decoupling of Light and Dark Reactions for Delayed on-Demand Solar Hydrogen Production. *Nat. Chem.* **2022**, *14* (3), 321–327. https://doi.org/10.1038/s41557-021-00850-8.

(9) te Velde, G.; Bickelhaupt, F. M.; Baerends, E. J.; Fonseca Guerra, C.; van Gisbergen, S. J. A.; Snijders, J. G.; Ziegler, T. Chemistry with ADF. *J. Comput. Chem.* **2001**, *22* (9), 931–967. https://doi.org/10.1002/jcc.1056.

(10) O'Boyle, N. M.; Banck, M.; James, C. A.; Morley, C.; Vandermeersch, T.; Hutchison, G. R. Open Babel: An Open Chemical Toolbox. *J Cheminform* **2011**, *3* (1), 33. https://doi.org/10.1186/1758-2946-3-33.

(11) Seritan, S.; Bannwarth, C.; Fales, B. S.; Hohenstein, E. G.; Isborn, C. M.; Kokkila-Schumacher, S. I. L.; Li, X.; Liu, F.; Luehr, N.; Snyder, J. W.; Song, C.; Titov, A. V.; Ufimtsev, I. S.; Wang, L.; Martínez, T. J. TeraChem : A Graphical Processing Unit -accelerated Electronic Structure Package for large-scale Ab Initio Molecular Dynamics. *WIREs Comput Mol Sci* **2021**, *11* (2), e1494. https://doi.org/10.1002/wcms.1494.

(12) Mardirossian, N.; Head-Gordon, M. Thirty Years of Density Functional Theory in Computational Chemistry: An Overview and Extensive Assessment of 200 Density Functionals. *Molecular Physics* **2017**, *115* (19), 2315–2372. https://doi.org/10.1080/00268976.2017.1333644.
